# Adherence, Switches, and Drug Spending After Angiotensin Receptor Blocker Recalls and Shortages

**DOI:** 10.1001/jamahealthforum.2025.4078

**Published:** 2025-11-26

**Authors:** Katherine Callaway Kim, Eric T. Roberts, Julie M. Donohue, Lindsay M. Sabik, Chester B. Good, Joshua W. Devine, Mina Tadrous, Katie J. Suda

**Affiliations:** 1 Department of Health Policy and Management, University of Pittsburgh School of Public Health, Pittsburgh, Pennsylvania; 2 Division of General Internal Medicine, Department of Medicine, University of Pittsburgh School of Medicine, Pittsburgh, Pennsylvania; 3 Division of General Internal Medicine, Department of Medicine, University of Pennsylvania Perelman School of Medicine, Philadelphia; 4 Deputy Editor, *JAMA Health Forum*; 5 Center for Value-Based Pharmacy Initiatives, University of Pittsburgh Medical Center (UPMC) Health Plan, Pittsburgh, Pennsylvania; 6 Department of Public Health, Des Moines University, Des Moines, Iowa; 7 Leslie Dan Faculty of Pharmacy, University of Toronto, Toronto, Ontario, Canada; 8 Women’s College Research Institute, Toronto, Ontario, Canada; 9 Center for Healthcare Evaluation, Research, and Promotion, VA Pittsburgh Healthcare System, Pittsburgh, Pennsylvania

## Abstract

**Importance:**

Angiotensin II receptor blockers (ARBs) are common treatments for hypertension, heart failure, and chronic kidney disease. From 2018 to 2019, hundreds of valsartan, losartan, and irbesartan products were recalled due to ingredient impurities.

**Objective:**

To estimate the impact of the 2018 to 2019 ARB shortages on medication adherence, switches to alternatives, and associated drug spending up to 18 months.

**Design, Setting, and Participants:**

This longitudinal cohort study with a difference-in-differences (DiD) analysis used pharmacy claims data from IQVIA’s all-payer Formulary Impact Analyzer dataset from July 2017 to January 2020, comprising prerecall users of valsartan, irbesartan, and losartan vs similar nonrecalled medications (other ARBs, angiotensin-converting enzyme inhibitors [ACEIs]). Analyses were conducted from November 2023 to October 2025.

**Exposures:**

Use of the recalled drugs (valsartan, irbesartan, and losartan) at baseline vs comparison antihypertensives (nonrecalled ARBs, ACEIs).

**Main Outcomes and Measures:**

Mean proportion of days covered for ARBs and ACEIs, switches to alternatives, medication gaps of 30 or more days, and associated drug spending (insurer and patient out-of-pocket costs).

**Results:**

For 13.8 million ARB users (median [IQR] age in 2018, 66 [56-74] years; 54.8% female) vs 23.4 million comparison drug users (median [IQR] age in 2018, 62 [54-72] years; 46.0% female), mean proportion of days covered changed by 0.55 percentage points (pp; 95% CI, 0.34-0.76 pp) within 18 months. Relative changes in gaps of 30 or more days, insurer drug spending, and patient out-of-pocket drug spending changed by less than 5% (relative changes of −2.5%, 0.6%, and 3.7%, respectively). ARB users experienced an increase in medication switches in the 90 days after the valsartan recall (DiD estimate: 8.46 pp; 95% CI, 8.30-8.63 pp; 229.0% relative increase). Smaller increases in switching occurred after the first irbesartan and first losartan recalls (DiD estimate: 1.20 pp; 95% CI, 1.12-1.27 pp; 32.4% relative increase). The proportion of individuals switching was greater among those with Medicare (DiD estimate: 9.49 pp; 95% CI, 9.28-9.72 pp; 256.8% relative increase) or third-party insurance (DiD estimate: 7.81 pp; 95% CI, 7.57-8.04 pp; 210.8% relative increase) vs Medicaid fee-for-service insurance (DiD estimate: 2.54 pp; 95% CI, 2.31-2.77 pp; 43.1% relative increase) or among customers paying with cash (DiD estimate: 3.42 pp; 95% CI, 3.22-3.61 pp; 87.1% relative increase).

**Conclusions and Relevance:**

This cohort study shows that access to alternatives may have mitigated gaps in treatment during the 2018 to 2019 ARB recalls and drug shortages. Potential disparate impacts among certain subgroups highlight the need for policies to mitigate financial and other systematic access barriers to receiving health care during drug shortages.

## Key Points

**Question:**

How did antihypertension medication use and drug spending change after shortages for angiotensin II receptor blockers (ARBs; valsartan, irbesartan, and losartan) from 2018 to 2019?

**Findings:**

In this cohort study of 13.8 million ARB users, mean adherence to antihypertension medications did not change within 18 months after the ARB recalls, though many users of recalled ARBs switched to alternatives within 90 days, with no associated increase in insurer or out-of-pocket drug spending. Increases in switching were lower among Medicaid fee-for-service beneficiaries and customers paying in cash vs those with Medicare or third-party insurance.

**Meaning:**

Switches to alternatives likely mitigated gaps in treatment during the ARB shortages, and differences by insurance highlight the need for policies to address systematic barriers to receiving health care during drug shortages.

## Teaser

This cohort study estimates the impact of the 2018 to 2019 angiotensin II receptor blocker shortages on medication adherence, switches to alternatives, and associated drug spending up to 18 months.

## Introduction

Angiotensin II receptor blockers (ARBs) are common medications to treat hypertension, heart failure, and chronic kidney disease.^1,2^ From 2018 to 2019, specific lots of 3 ARBs—valsartan, losartan, and irbesartan—were recalled due to ingredient impurities with N-nitrosamines, a probable human carcinogen.^3,4^ Although cancer risk was low, hundreds of products were recalled, resulting in global drug shortages.^5^

During the 2018 to 2019 ARB shortages, the US Food and Drug Administration (FDA) recommended that patients continue to take their prescribed medications until they could transition to alternatives. Studies from Canada and Europe suggest that many patients transitioned to other, similar drugs (eg, angiotensin-converting enzyme inhibitors [ACEIs]).^6,7^ However, qualitative studies in the US suggest that the recalls lessened some individuals’ confidence in the safety of antihypertensives.^8^ Transitions to alternatives could impose time, cost, and other burdens by requiring multiple health care visits to determine dosages and monitor adverse events.^4,9^ In 1 Canadian study, 1 in 10 valsartan users failed to fill any ARB or ACEI prescription within 6 months.^6^ Previous US studies suggest that switches to available alternatives may have prevented gaps in access and subsequent clinical harms for patients with commercial or Medicare Advantage insurance.^5,10,11^ However, the longer-term consequences of ARB shortages, including variations among differently insured populations—such as US individuals with low income who are enrolled in Medicaid or uninsured and other individuals who pay for prescriptions with cash—are unknown.

The impact of the 2018 to 2019 ARB shortages on drug spending is also unknown. Shortages for ARB generics may have forced patients to transition to branded drugs.^4,12^ Shortage-related spikes in out-of-pocket drug spending may have been associated with worse adherence,^13^ especially for patients with low income or who are underinsured and may be responsible for a greater proportion of cost sharing.^14,15^

The objective of this study was to characterize longer-term (18-month) changes in medication utilization and drug spending attributable to the 2018 to 2019 ARB recalls and drug shortages. We used all-payer data from a national pharmacy database to compare longitudinal changes in outcomes for US patients using valsartan, losartan, and irbesartan prerecall vs changes among unexposed patients using similar nonrecalled drugs (ie, ACEIs, nonrecalled ARBs like candesartan).

## Methods

### Data Source and Study Population

We conducted a national longitudinal study of medication users in IQVIA’s all-payer Formulary Impact Analyzer dataset (2017-2020), comprising more than 60% of prescription fills dispensed in US retail, long-term care, and mail order pharmacies. The IQVIA extract included ARBs, ACEIs, other renin-angiotensin agents, diuretics, α- and β-adrenergic blockers, and calcium channel blockers (eTable 1 in Supplement 1). We included final claims for both completed prescription fills and rejected prescription fills (eg, due to missing information or prior authorization requirements). Because IQVIA is a prescription-only dataset, it does not contain diagnosis or procedure codes, clinical outcomes like blood pressure, nor complete information on demographics like race and ethnicity.

The study population comprised prevalent baseline users of the recalled ARBs (valsartan, losartan, and irbesartan) or similar comparison drugs that did not go into shortage (ACEIs, nonrecalled ARBs like candesartan [eTable 1 in Supplement 1]). We restricted to established users by requiring medication possession in the quarter prerecall and at least 2 prescription fills in the baseline year. We categorized individuals as ARB users if their most recent prerecall prescription fill was for valsartan, losartan, or irbesartan, including combination products. Consistent patient identifiers allowed assessment of longitudinal changes in medication use.

The University of Pittsburgh approved this study as nonhuman participant research; therefore, informed consent was not required. We followed Strengthening the Reporting of Observational Studies in Epidemiology (STROBE) reporting guidelines.

### Study Period

The study period spanned the 4 quarters before to 6 quarters after the first recall for valsartan on July 13, 2018 (eFigure 1 in Supplement 1). Data were aggregated into 90-day quarters. The valsartan recall represented a sentinel event that revealed safety concerns in the ingredient supply chain. Some losartan and irbesartan users may have proactively switched to alternatives before these drugs were recalled on October 30, 2018, and November 9, 2018, respectively.^3,9^ The postperiod therefore comprised 2 intervals: (1) the 90-day period after valsartan recall (July 13, 2018, to October 10, 2018) and (2) the subsequent 5 quarters containing all 3 shortages (October 11, 2018, to January 3, 2020).

### Medication Use Outcomes

Switches to alternatives and changes in medication adherence are an important symptom of drug shortages that may increase time, financial, and other logistical barriers to accessing needed health care, especially for those who are uninsured or underinsured. To capture the overall impact of the 2018 to 2019 ARB shortages on medication use, the primary adherence measure was the proportion of days covered (PDC) for any recalled ARB or comparison drug. PDC was defined as the number of days covered divided by the total number of days per quarter. Patients were censored on the last day covered by any recalled ARB or comparison drug fill. Because the present dataset was sourced from pharmacies, it did not contain days enrolled in insurance. The PDC measure ranged from 0 to 100 percentage points (pp) and accounted for both previous and current prescription fills; we did not adjust for early prescription fills. To assess factors associated with PDC, secondary outcomes included the proportion of patients with medication switches (any drug fill for a nonindex medication that overlapped or occurred <30 days after an index fill ran out^7^), 30-day or more gaps between prescription fills (representing approximately 1 missed prescription), and the average number of days between prescription fills to assess potential delays in access among those who did not miss any prescription fills.

### Drug-Spending Outcomes

We examined both insurer spending and patient out-of-pocket spending for ARBs or comparison drugs. Insurer spending reflected negotiated amounts. Cost sharing in IQVIA was defined as the amount paid after all secondary payments such as coupons were applied; separate variables for deductibles, coinsurance, or co-payments did not exist.^16^ To understand potential mechanisms for changes in spending, we assessed the average number of generic prescription fills and rejected prescription fills per patient.

### Covariates

Variables in IQVIA included age, gender, US region, insurance type (Medicare, third party, Medicaid fee-for-service [Medicaid managed care is likely classified as third party], or cash/self-pay), and 3-level zip code. We categorized patient zip codes as predominantly metropolitan or nonmetropolitan based on 2010 rural-urban commuting areas (eTable 1 in Supplement 1). Each patient was assigned to a single prescriber specialty based on the prescriber associated with the most prerecall prescription fills (eTable 1 in Supplement 1). We categorized patients into low (<50 pp), moderate (51-79 pp) or high (≥80 pp) adherence groups based on their average quarterly PDC during the baseline year.^17^

### Statistical Analysis

Descriptive analyses compared baseline characteristics for ARB vs comparison drug users, with absolute standardized differences less than 0.2 used to deem groups well balanced. Linear difference-in-differences (DiD) models were estimated for the intention-to-treat differential changes in outcomes among all baseline prerecall ARB users (treatment group) vs all baseline comparison drug users. We estimated separate effects for (1) the 90-day period after valsartan’s recall (July 13, 2018, to October 10, 2018) and (2) all subsequent quarters after the irbesartan and losartan shortages (October 11, 2018, to December 5, 2019). Due to large sample sizes (>35 million), individual-level models for the full sample were time and computing prohibitive. We, therefore, first aggregated the individual-level data to the subgroup-quarter level, with groups defined by the covariates previously described. Linear DiD models were estimated using these aggregated data. Analytic weights reflected the size of each stratum relative to the total population. We conducted a sensitivity analysis using individual-level data for a 20% random sample. The key assumption was that outcome trends among ARB users would have continued the same trajectory as comparison drug users if the shortages had not occurred.^18^ To evaluate the plausibility of this assumption, we tested for parallel trends prerecall. Statistical significance was assessed at *P* < .05 using 2-sided tests with no adjustments for multiple testing. Analyses were conducted using Stata, version 17.0 (StataCorp); SAS, version 9.4 (SAS Institute); and RStudio, version 2024.04.2 (Posit) from November 2023 to October 2025.

### Sensitivity Analyses

Several sensitivity analyses assessed methodological assumptions and the robustness of results. First, we repeated the DiD analysis using individual patient-quarter–level data from a random 20% sample. Second, we relaxed the requirement for medication possession in the quarter prerecall; this mitigated potential regression-to-the-mean concerns from restricting to active users. Third, we removed ACEI users and individuals who filled 75% or more of their prerecall prescriptions in long-term care pharmacies, given potential systematic differences. Fourth, we used 15, 45, 60, and 90 days (vs 30 days) to define medication gaps.^19^ Fifth, we respecified the models using a comparative interrupted time series approach to test robustness against violations of the DiD parallel pretrends assumption.^18^

## Results

### Characteristics of ARB and Comparison Drug Users

There were 13.8 million users of valsartan, losartan, and irbesartan (recalled ARBs) and 23.4 million comparison drug users who met inclusion criteria (Table 1 and eFigure 2 in Supplement 1). Among recalled ARB users, 72.2% had losartan as their most recent prescription fill prerecall, followed by 22.9% with valsartan and 4.9% with irbesartan. Of comparison drug users, 94.5% filled prescriptions for ACEIs vs 5.5% for nonrecalled ARBs. Based on standardized differences (>0.20), recalled ARB users vs comparison drug users were older (median [IQR] age, 66 [56-74] years vs 62 [54-72] years), more likely to be female (54.8% vs 46.0%), more likely to be enrolled in Medicare (44.5% vs 38.2%), and used more antihypertensives at baseline (≥4 drug classes: 8.7% vs 6.0%); additionally, a higher proportion of recalled ARB users received prescription fills from cardiovascular disease specialists (11.1% vs 8.5%) or nephrologists (1.8% vs 1.2%) (Table 1). Characteristics of individuals in the IQVIA dataset were consistent before and after the recall (eTable 2 in Supplement 1).

**Table 1.** Baseline Characteristics of Established Angiotensin II Receptor Blocker (ARB) and Comparison Drug Users, July 2017 to June 2018

Characteristic	No. (%)		Standardized difference
	Users of recalled ARBs at baseline	Users of comparison drug at baseline	
Total	13 755 251 (100)	23 426 296 (100)	NA
Most recent prerecall prescription fill			
Valsartan	3 147 530 (22.9)	NA	NA
Irbesartan	671 438 (4.9)	NA	
Losartan	9 936 283 (72.2)	NA	
ACEI	NA	22 147 622 (94.5)	
Nonrecalled ARB^a^	NA	1 278 674 (5.5)	
Age in 2018, median (IQR), y	66 (56-74)	62 (54-72)	0.18
Age group in 2018, y			
18-19	7069 (0.1)	26 710 (0.1)	0.18
20-29	77 126 (0.6)	217 469 (0.9)	
30-39	373 762 (2.7)	930 796 (4.0)	
40-49	1 248 347 (9.1)	2 653 751 (11.3)	
50-64	4 729 740 (34.4)	8 975 948 (38.3)	
65-79	5 384 236 (39.1)	8 092 124 (34.5)	
≥80	1 934 971 (14.1)	2 529 498 (10.8)	
Gender			
Female	7 533 747 (54.8)	10 773 448 (46.0)	0.18
Male	6 221 504 (45.2)	12 652 848 (54.0)	
US region			
Northeast	2 400 137 (17.4)	3 876 451 (16.5)	0.10
Midwest	2 719 913 (19.8)	5 348 442 (22.8)	
South	5 680 747 (41.3)	9 487 052 (40.5)	
West	2 520 184 (18.3)	4 032 751 (17.2)	
Missing/unknown	434 270 (3.2)	681 600 (2.9)	
Patient residential location based on 3-level zip code			
<50% Metropolitan by population	1 389 755 (10.1)	2 858 347 (12.2)	0.07
≥50% Metropolitan by population^b^	11 017 935 (80.1)	18 448 542 (78.8)	
Missing/unknown	1 347 561 (9.8)	2 119 407 (9.0)	
Prerecall PDC, median (IQR)	91.1 (76.6-97.1)	90.2 (75.0-96.8)	0.07
Prerecall PDC category			
Low (<50 pp)	621 186 (4.5)	1 300 660 (5.6)	0.05
Moderate (51-79 pp)	3 436 714 (25.0)	6 157 964 (26.3)	
High (≥80 pp)	9 697 351 (70.5)	15 967 672 (68.2)	
Most common prerecall prescription fill location			
Retail pharmacy	11 627 233 (84.5)	20 119 469 (85.9)	0.08
Mail-order pharmacy	1 466 405 (10.7)	2 203 769 (9.4)	
Long-term care facility	108 819 (0.8)	203 726 (0.9)	
Unknown/mixed/missing	552 794 (4.0)	99 332 (3.8)	
Insurance type on last prerecall prescription fill			
Medicare^c^	6 122 101 (44.5)	8 940 581 (38.2)	0.24
Third party^d^	7 306 139 (53.1)	13 221 684 (56.4)	
Medicaid fee-for-service	139 892 (1.0)	383 420 (1.6)	
Cash/self-pay	187 119 (1.4)	880 611 (3.8)	
Primary prescriber specialty prerecall^e^			
Internal medicine or other general practice	9 105 323 (66.2)	5 211 694 (64.9)	0.21
Physician assistant or nurse practitioner	2 127 742 (15.5)	4 587 815 (19.6)	
Cardiovascular disease	1 528 836 (11.1)	1 994 061 (8.5)	
Nephrology	249 122 (1.8)	283 479 (1.2)	
Diabetes, endocrine, or other metabolic disease	151 667 (1.1)	248 440 (1.1)	
Other specialty	499 340 (3.6)	894 935 (3.8)	
Missing/unknown	93 221 (0.7)	205 872 (0.9)	
Prerecall antihypertension medication use category			
1 Drug class	4 835 190 (35.2)	10 254 229 (43.8)	0.16
2 Drug classes	4 713 553 (34.3)	7 664 570 (32.7)	
3 Drug classes	3 013 280 (21.9)	4 106 274 (17.5)	
≥4 Drug classes	1 193 228 (8.7)	1 401 223 (6.0)	
Prerecall use of other antihypertension drug classes^f^			
Calcium channel blockers	4 554 287 (33.1)	5 624 094 (24.0)	0.20
Diuretics	4 053 268 (29.5)	6 110 257 (26.1)	0.08
Adrenergic blockers	5 416 054 (39.4)	8 045 544 (34.3)	0.10
Other renin-angiotensin agents	32 929 (0.2)	29 155 (0.1)	0.03
Other vascular agents	406 030 (3.0)	412 819 (1.8)	0.08

Abbreviations: ACEI, angiotensin-converting enzyme inhibitor; NA, not applicable; PDC, proportion of days covered; pp, percentage points.

^a^ Nonrecalled ARBs included azilsartan, candesartan, eprosartan, olmesartan, and telmisartan, including combination products (eTable 1 in Supplement 1).

^b^ Areas were defined as predominantly metropolitan if 50% or more of the population resided in zip codes classified as 2010 rural-urban commuting areas 1 (metropolitan area, core), 2 (metropolitan area, high commuting) or 3 (metropolitan area, low commuting).

^c^ Medicare insurance type included both Medicare and Medicare Part D.

^d^ Third party insurance included coverage from an employer, Medicaid managed care, and individual market plans. We did not have access to reliable variables to distinguish between these types.

^e^ Patients were assigned a single primary prescriber based on the most prescription fills in the prerecall period (July 18, 2017, to July 12, 2018). See eTable 1 in Supplement 1 for codes used to define each prescriber specialty.

^f^ Specific drugs included in each antihypertension class can be found in eTable 1 in Supplement 1.

### Unadjusted Outcomes

There were 16 million to 20 million prescription fills per quarter from July 2017 to January 2020 among recalled ARB users and 26 million to 38 million prescription fills among comparison drug users. There was also a sudden decrease in valsartan prescription fills among ARB users in July 2018, with subsequent increases in prescription fills for other ARBs; these changes were not observed among the comparison group (eFigure 3 in Supplement 1).

**Figure 1.**
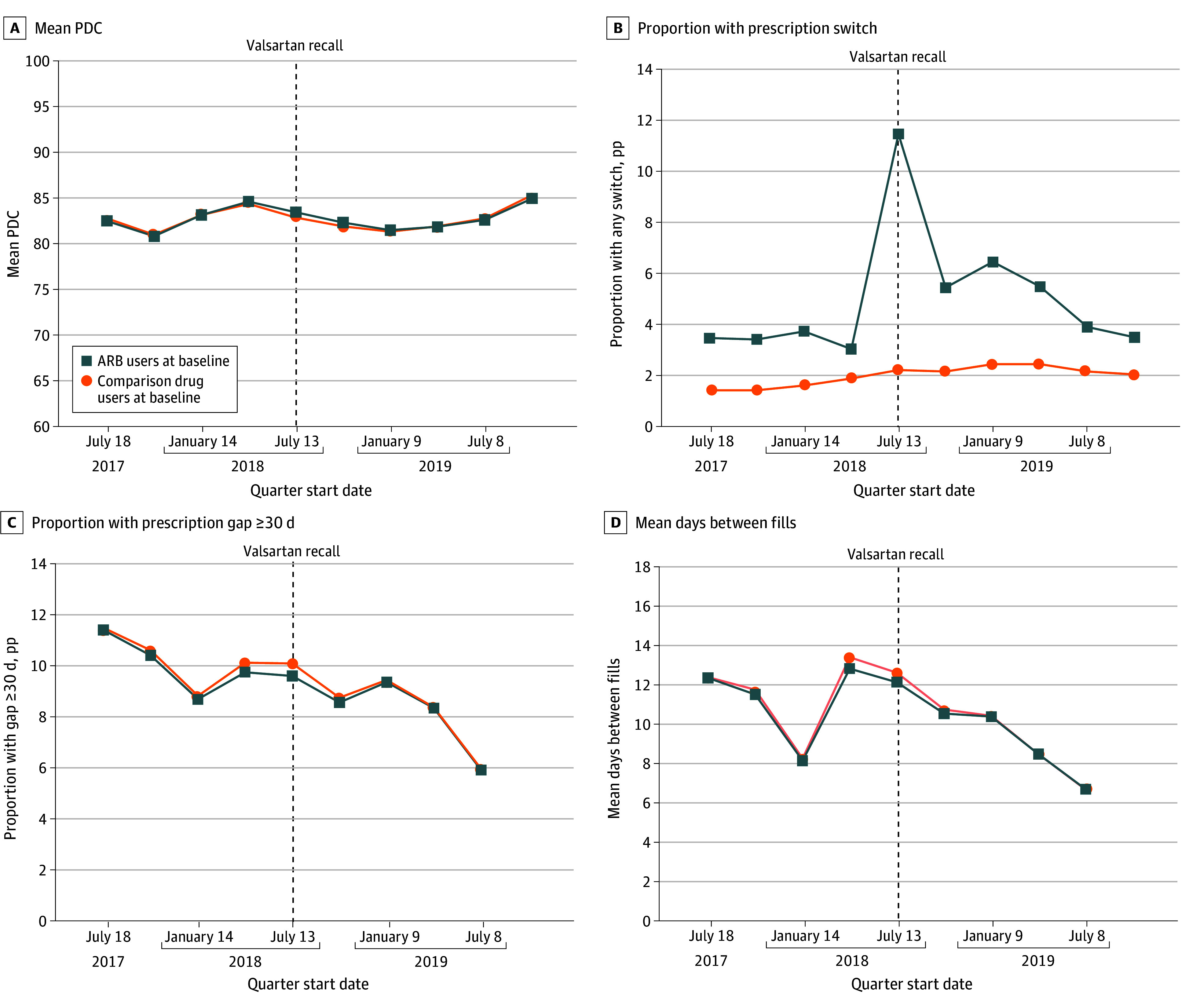
Standardized Trends in Medication Use Outcomes for Users of Recalled Angiotensin II Receptor Blockers (ARBs) vs Comparison Drugs, Before and After Recall Quarters were defined as 90-day intervals indexed to the first ARB recall for valsartan on July 13, 2018. Rates were standardized to the population distributions for the following prerecall characteristics: age group (18-19, 20-29, 30-39, 40-49, 50-64, 65-79, ≥80 years), female gender, prerecall fill location (retail, long-term care, mail order, or unknown/mixed), prerecall antihypertension medication use category (1, 2, 3, or ≥4 drug classes), prerecall proportion of days covered (PDC) category (<50, 51-79, ≥80 percentage points [pp]), primary prescriber specialty, US geographic region, patient metropolitan residential location, and insurance type (Medicare, third party, Medicaid fee-for-service, or cash/self-pay).

A high (>85 pp) baseline PDC to the drugs of interest for both groups was observed and remained high postrecall (Figure 1A). The proportion of patients with medication gaps of 30 or more days and mean days between fills decreased over time and did not differ between groups (Figure 1C and D). However, an increase in users of recalled ARBs who switched medications in the quarter after the valsartan recall was observed (July 13, 2018, to October 10, 2018), with another peak in switching approximately 2 quarters later (January 9, 2019, to April 8, 2019; Figure 1B).

**Figure 2.**
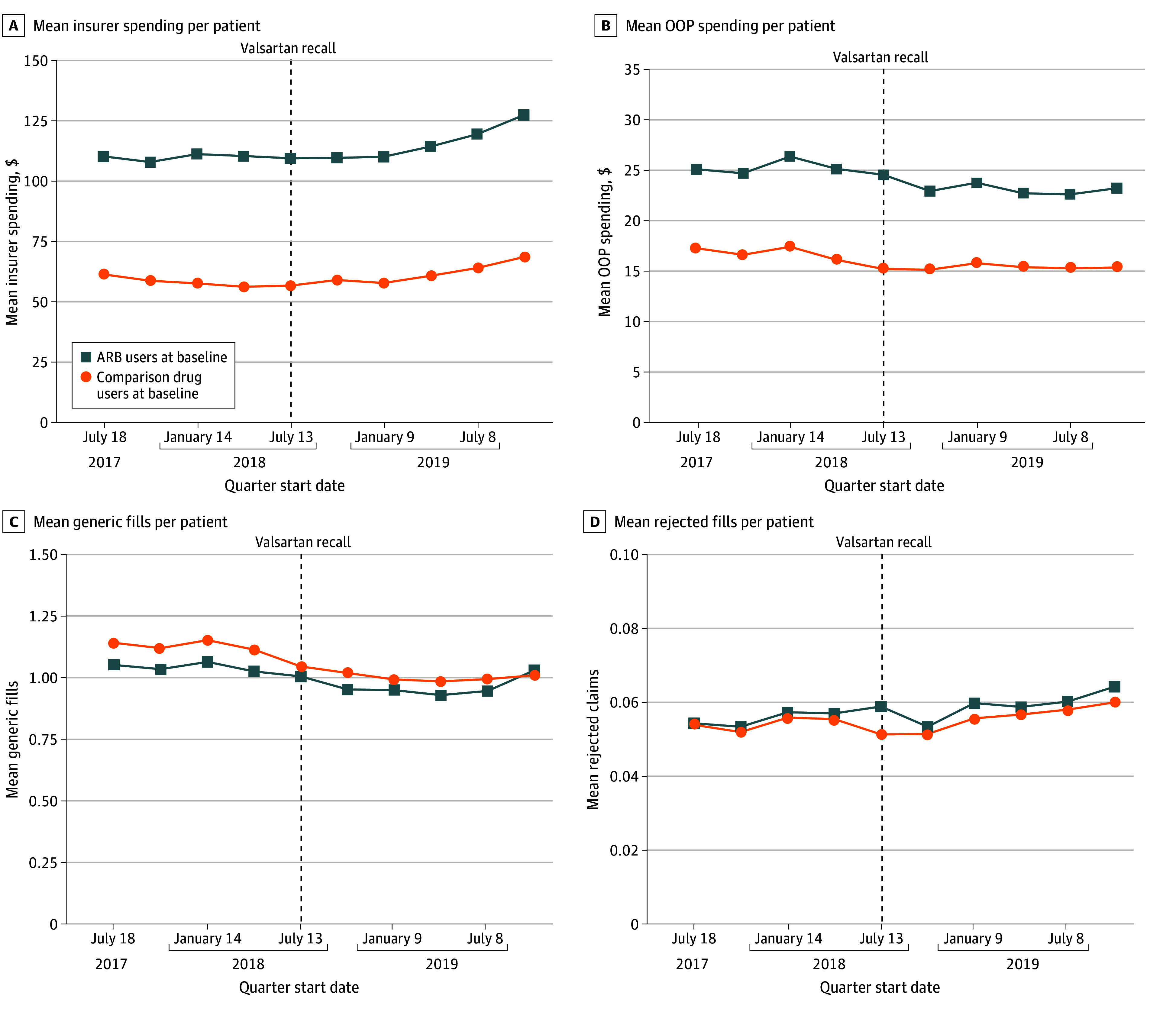
Standardized Trends in Drug Spending Outcomes for Established Users of Angiotensin II Receptor Blockers (ARBs) vs Comparison Drugs, Before and After Recall Quarters were defined as 90-day intervals indexed to the first ARB recall for valsartan on July 13, 2018. Rates were standardized to the population distributions for the following prerecall characteristics: age group (18-19, 20-29, 30-39, 40-49, 50-64, 65-79, ≥80 years), female gender, prerecall fill location (retail, long-term care, mail order, or unknown/mixed), prerecall antihypertension medication use category (1, 2, 3, or ≥4 drug classes), prerecall proportion of days covered category (<50, 51-79, ≥80 percentage points), primary prescriber specialty, US geographic region, patient metropolitan residential location, and insurance type (Medicare, third party, Medicaid fee-for-service, or cash/self-pay). Population weights used for the standardization are shown in eTable 2 in Supplement 1. OOP indicates out of pocket.

Similar trends in insurer drug spending, patient out-of-pocket spending, and generic prescription fills across groups were observed (Figure 2A-C). There was a small increase in rejected prescription fills among users of recalled ARBs in the quarter after the valsartan recall (Figure 2D).

### Adjusted DiD Results

Event-study plots supported the DiD parallel pretrends assumption (eFigures 4 and 5 in Supplement 1). Consistent with unadjusted findings, relative adjusted DiD estimates in PDC, gaps of 30 or more days, and the days between fills were less than 5% per quarter postrecall. The proportion of users of recalled ARBs with medication switches increased by 8.46 pp (95% CI, 8.30-8.63 pp), a 229.0% relative increase, in the first quarter after valsartan’s recall. Smaller increases in switches over the subsequent 5 quarters were observed (per quarter: 1.20 pp; 95% CI, 1.12-1.27 pp; 32.4% relative increase). There were no substantial differential increases in insurer drug spending, patient out-of-pocket spending, or generic prescription fills (relative changes, <5%). Rejected prescription fills increased by 0.007 (95% CI, 0.007-0.008) per patient in the quarter after the valsartan recall, a 11.9% relative increase compared to a baseline rate of 0.061 (Table 2; full regression output in eTables 3 and 4 in Supplement 1). Of rejected prescription fills among users of recalled ARBs in the quarter after valsartan’s recall, 54.6% were for refill restrictions, 23.0% were for step-edit requirements, and 6.2% were due to the product not being covered. There were not differences in reasons for rejection between the study groups or before vs after recalls (eTable 5 in Supplement 1).

**Table 2.** Adjusted Differential Changes in Outcomes for Established Angiotensin II Receptor Blocker (ARB) vs Comparison Drug Users, Before and After Recall

Outcome	Mean (SD) prerecall use per quarter (July 18, 2017, to July 12, 2018)			Difference-in-differences estimates^a^					
				First quarter of valsartan recall (July 13, 2018, to October 10, 2018)			After first irbesartan and losartan recalls (October 11, 2018, to January 3, 2020)		
	Users of recalled ARBs at baseline	Users of comparison drugs at baseline	Difference	Coefficient (95% CI)	*P* value	Relative change, %^b^	Coefficient (95% CI)	*P* value	Relative change, %^b^
Mean PDC per patient-quarter, pp	85.62 (22.73)	84.60 (23.48)	1.02	0.55 (0.34 to 0.76)	<.001	0.6	−0.17 (−0.37 to 0.02)	.08	−0.2
% With prescription switch	3.70 (18.87)	1.59 (12.52)	2.10	8.46 (8.30 to 8.63)	<.001	229.0	1.20 (1.12 to 1.27)	<.001	32.4
% With prescription gap ≥30 d	9.45 (29.25)	10.24 (30.32)	−0.79	−0.24 (−0.45 to −0.03)	.03	−2.5	0.35 (0.14 to 0.56)	.001	3.7
Mean days between fills	10.61 (31.14)	11.56 (33.12)	−0.94	−0.28 (−0.46 to −0.10)	<.001	−2.6	0.51 (0.29 to 0.72)	<.001	4.8
Mean payer prescription spending per patient-quarter, $	122.60 (289.85)	61.55 (172.25)	61.04	0.79 (−1.05 to 2.64)	.40	0.6	2.23 (1.02 to 3.44)	<.001	1.8
Mean OOP prescription spending per patient-quarter, $	25.32 (52.62)	18.41 (46.26)	6.91	0.95 (0.56 to 1.33)	<.001	3.7	−0.53 (−0.76 to −0.30)	<.001	−2.1
Generic prescription fills	1.13 (1.17)	1.21 (1.16)	−0.09	0.05 (0.04 to 0.06)	<.001	4.7	0.05 (0.04 to 0.05)	<.001	4.3
Rejected prescription fills	0.061 (0.26)	0.058 (0.25)	0.003	0.007 (0.007 to 0.008)	<.001	11.9	0.002 (0.002 to 0.002)	<.001	3.2

Abbreviations: OOP, out-of-pocket; PDC, proportion of days covered; pp, percentage points.

^a^ Difference-in-differences estimates for the intention-to-treat differential change in outcomes for baseline ARB vs comparison drug users. Models were adjusted for age group (18-19, 20-29, 30-39, 40-49, 50-64, 65-79, ≥80 years), female gender, prerecall prescription fill location (retail, long-term care, mail order, or unknown/mixed), prerecall antihypertension medication use category (1, 2, 3, or ≥4 drug classes), prerecall medication possession ratio category (<50, 51-79, ≥80), primary prescriber specialty, US geographic region, patient metropolitan residential location, and insurance type (Medicare, third party, Medicaid fee-for-service, or cash/self-pay). Full model output can be found in eTables 2 and 3 in Supplement 1.

^b^ Relative changes were calculated as the difference-in-differences estimate, divided by the mean quarterly use among ARB users in the prerecall period (July 18, 2017, to July 12, 2018).

### Sensitivity Analyses

Models fit on individual-level data for a 20% random sample were consistent with the main findings (eTables 6 and 7 in Supplement 1), as were analyses with different cohort inclusion criteria (eTables 8 and 9 in Supplement 1). There were no differential changes in 15-, 45-, 60-, or 90-day (vs 30-day) gaps (eTable 10 in Supplement 1). An interrupted time series approach resulted in attenuated differential increases in switching after the valsartan recall (6.72 pp; 95% CI, 6.53-6.91 pp; 172% relative increase; eTable 11 in Supplement 1).

### Post Hoc Analyses by Patient Characteristics

To understand differential impacts on different patient populations, an exploratory analysis was conducted and stratified by patient characteristics, which may be associated with more difficulty in switching medications during drug shortages. ARB users who switched medications in the 90 days after the valsartan recall were more likely than those who did not to be baseline users of this drug (88.8% vs 12.9%) and had higher baseline PDC (93.1% vs 90.5%) (eTable 12 in Supplement 1). Consistent with the greater total number of recalled lots of valsartan, switches increased by 44.0 pp (95% CI, 43.5-44.5 pp) among valsartan users in the quarter after the valsartan recall (July 2018) vs 5.8 pp (95% CI, 5.6-6.0 pp) among irbesartan users and 0.2 pp (95% CI, 0.1-0.2 pp) among losartan users after these drugs went on shortage (October 2018; eFigure 6 and eTable 13 in Supplement 1). Although rejected prescription fills remained rare (3 per 100 patients per quarter), rejections increased by 49.9% among valsartan users in the first quarter postrecall (eFigure 7 and eTable 14 in Supplement 1). During this quarter, compared to prerecall, a greater proportion of rejected prescription fills for baseline valsartan users were for missing claim information (19.4% vs 8.7%) or products not being covered (15.1% vs 5.5%) (eFigure 15 in Supplement 1). Changes in rejected prescription fills among baseline users of irbesartan or losartan were not observed (eFigure 7 and eTables 14 and 15 in Supplement 1).

**Figure 3.**
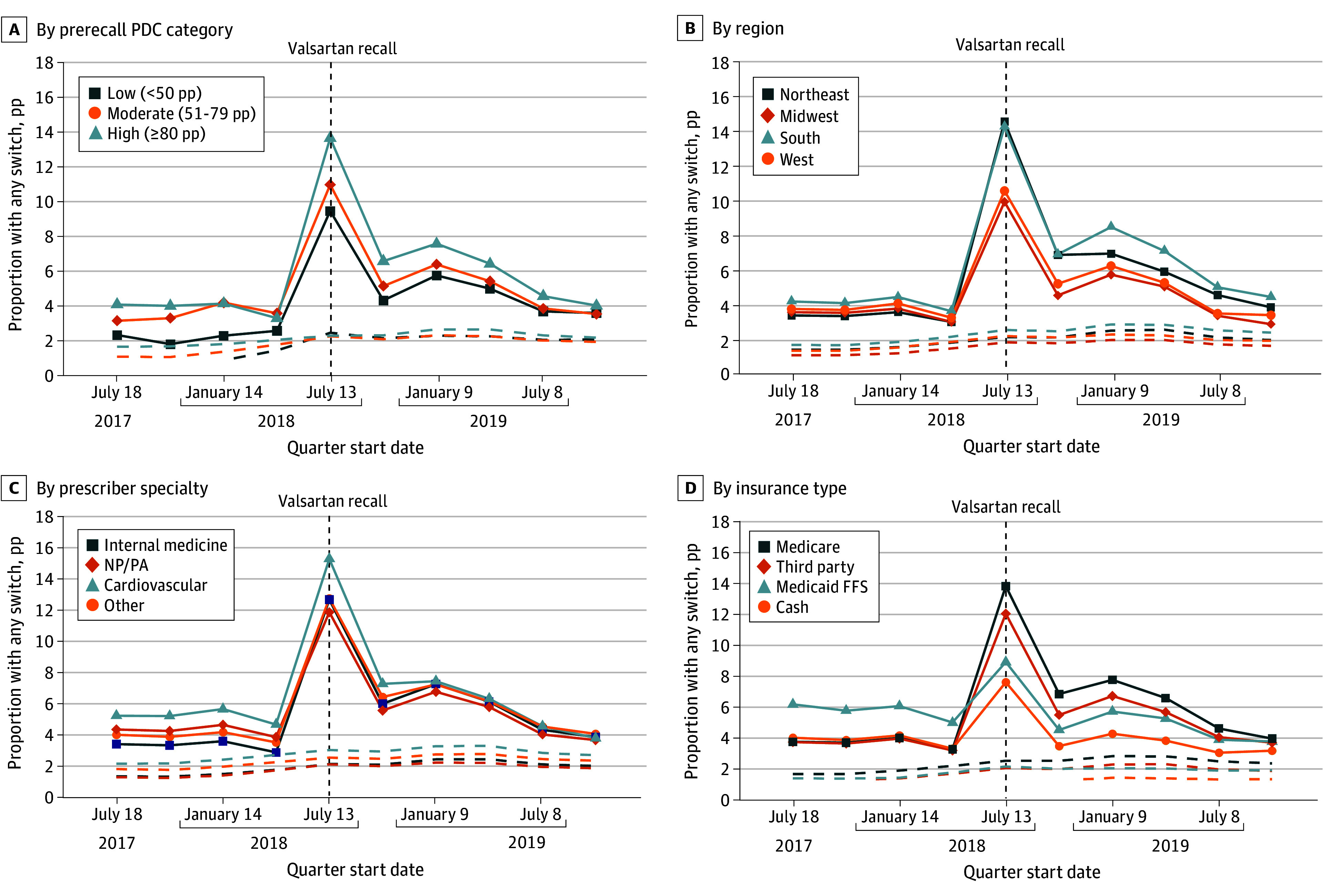
Unadjusted Trends in Individuals With Prescription Switch, Post Hoc Sensitivity Analysis by Baseline Medication Possession Ratio, Region, Prescriber Specialty, and Insurance Type Quarters were defined as 90-day intervals indexed to the first angiotensin II receptor blocker recall for valsartan on July 13, 2018. Solid lines represent the proportion of angiotensin II receptor blocker users with any switch per quarter. Dashed lines represent trends for comparison drug users. FFS indicates fee-for-service; NP/PA, nurse practitioner/physician assistant; PDC, proportion of days covered; pp, percentage points.

Higher increases in switching were observed among individuals with high or moderate prerecall PDC, in the Northeast and South, and for patients who received prescription fills from internal medicine physicians or cardiovascular disease specialists (Figure 3A-C). In contrast, switching was lower for Medicaid fee-for-service beneficiaries and customers paying with cash (Figure 3D). Increases in switching in the quarter after the valsartan recall were 2.54 pp (95% CI, 2.31-2.77 pp; 43.1% relative increase) among Medicaid fee-for-service beneficiaries and 3.42 pp (95% CI, 3.22-3.61 pp; 87.1% relative increase) among customers paying with cash vs 9.49 pp (95% CI, 9.27-9.72 pp; 256.8% relative increase) and 7.81 pp (95% CI, 7.57-8.03 pp; 210.8% relative increase) for Medicare and third-party beneficiaries, respectively (eTable 16 in Supplement 1).

## Discussion

In this national study, medication switches increased immediately after valsartan was recalled, with no substantial changes in PDC and insurer or patient out-of-pocket drug spending. Despite the overall null findings on gaps in medication use and costs, we did see evidence of potentially disparate impacts among Medicaid fee-for-service beneficiaries and customers paying with cash, including lower switching rates. Effects were concentrated among valsartan users, consistent with a greater total number of recalled lots of this ARB. Unlike other recent generic shortages for which therapeutic substitutes were limited, availability of alternatives during the 2018 to 2019 ARB recalls may have mitigated financial or logistical barriers to access.

These results are consistent with those from Desai et al,^5^ who found that population-level prescription fills for nonvalsartan ARBs increased among US commercially insured beneficiaries in the 8 months after the valsartan recall, and Eworuke et al,^7^ who observed an increase in switching from valsartan to other ARBs in the US FDA Sentinel System, Canada, and Denmark. This study adds to this literature by assessing patient-level switching against a comparison cohort of individuals using nonrecalled drugs at baseline. We also evaluated differences by drug and patient characteristics, which may be associated with greater difficulty in accessing health care during drug shortages (eg, insurance type, out-of-pocket spending). The observed increase in medication switches within 90 days after the valsartan recall in this study likely prevented longer-term gaps in access. However, actual adherence to taking filled medications during drug shortages cannot be measured in pharmacy claims and is an important area of future research.

To our knowledge, this study is the first to find no observable increases in insurer and out-of-pocket drug spending during the 2018 to 2019 ARB shortages. Previous analyses suggest that drug shortages are associated with increased wholesale drug prices.^20^ However, depending on a patient’s insurance, increases in wholesale price may not always translate to changes in out-of-pocket spending. The finding of no increase in patient spending during the 2018 to 2019 ARB shortages contrasts with a previous study that found increased switches from a generic chemoprotective drug on shortage (leucovorin) to its branded counterpart (levoleucovorin).^12^ Unlike supply chains for drugs used to treat cancer, multiple therapeutic substitutes were available within the ARB class, many of which were generics. Redundancy at the drug-class level may have allowed many patients to switch to similar drugs, with likely similar insurance coverage. As policymakers consider solutions to drug shortages, increased competitive and available supply within therapeutic classes of public health importance could protect patients from clinical and financial harms during unexpected supply chain shocks.^21,22^

One key finding of our research was a lower rate of switching postrecall among Medicaid fee-for-service beneficiaries and customers paying with cash. The findings are consistent with prior studies in that many ARB users switched to alternative medications shortly after the shortages were announced.^5,7,11^ However, we lacked information on enrollees in Medicaid managed care organizations, which cover more than 70% of Medicaid beneficiaries, as well as data on race and ethnicity. Future research is needed to understand the interactive effects of race and ethnicity, baseline comorbidity, adherence, and insurance type, as individuals with greater socioeconomic and medical vulnerability may experience greater difficulty switching to alternative medications.

Future policies should consider all potential systematic, financial, and other logistical barriers to switching during drug shortages. Example policies include waiving co-payments for brand-name alternatives when a generic is on shortage. Furthermore, during the 2018 to 2019 ARB recalls, Veterans Affairs Pharmacy Benefit Management mailed letters that provided additional guidance to affected patients.^23^ Promotion of shared decision-making between prescribers and community pharmacists could increase flexibility for therapeutic substitutions at the point of scale. Policies that increase information available to community pharmacists and patients (eg, inclusion of lot numbers on stock containers) could decrease confusion and mitigate logistical and time barriers to switching during drug recalls. Assessment of these and other specific policies is beyond this study’s scope but are an important avenue for future research.

### Limitations

There were several limitations to this study. First, we did not have access to diagnosis codes to identify patients using the drugs of interest for treatment of hypertension vs heart failure or chronic kidney disease, nor clinical outcomes like blood pressure. Second, we lacked data on individuals enrolled in Medicaid managed care organizations, which cover a majority of Medicaid beneficiaries, underscoring a need for future research to examine the consequences of drug recalls among this population. Third, although PDC is a preferred method for measuring adherence in pharmacy claims, it does not capture medications that were filled but never consumed. It is also possible that patients followed FDA recommendations to continue taking their recalled medications until alternatives were available, switched to other antihypertensives (eg, calcium channel blockers), disenrolled from insurance, or filled medications in pharmacies that were not included in IQVIA. Previous population-level studies in Canada and Europe suggest little to no change in use of non-ARB, non-ACEI antihypertensives after the valsartan recall.^7,24^ Characteristics of patients with any ARB or ACEI fill over time were similar in this study, suggesting a lack of differential dropout from the IQVIA dataset. Finally, we described the impact of 1 event (the 2018 to 2019 ARB shortages) on medication use and spending. The finding of no major impact on these outcomes may not generalize to other shortages, including subsequent recalls for other classes of medications due to the same ingredient impurity.^25^

## Conclusions

This national cohort study found no changes in PDC and insurer or patient out-of-pocket drug spending in the 18 months after the valsartan, losartan, and irbesartan recalls and drug shortages. Many patients switched to available alternatives. However, we observed disparities in switching rates among certain subgroups. As policymakers consider solutions to drug shortages, developing policies that ease or improve switches to low-cost, alternative generics could increase access and mitigate downstream harm for everyone.

## Notes

Supplement 1. **eFigure 1. **Definition of Study Period **eTable 1. **Variable Definitions **eFigure 2. **Cohort Selection Diagram **eTable 2. **Baseline Characteristics of Patients with Any ARB or Comparison Drug Fill in IQVIA Dataset, by Study Month **eFigure 3. **Medication Fills for Established ARB vs. Comparison Drug Users, July 2017 – December 2019, by Drug Class **eFigure 4. **Adjusted Event-Study Plots for Differential Changes in Medication Utilization Outcomes for Established ARB vs. Comparison Drug Users, Pre- and Post-Recall **eFigure 5. **Adjusted Event-Study Plots for Differential Changes in Drug Spending Outcomes for Established ARB vs. Comparison Drug Users, Pre- and Post-Recall **eTable 3. **Full Difference-in-Difference Models for Adjusted Changes in Medication Use Outcomes Post- vs. Pre-Recall, Established ARB vs. Comparison Drug Users **eTable 4. **Full Difference-in-Difference Models for Adjusted Changes in Drug Spending Outcomes Post- vs. Pre-Recall, Established ARB vs. Comparison Drug Users **eTable 5. **Reasons for Rejected Fills, by Period and Study Group **eTable 6. **Baseline Characteristics of Established ARB and Comparison Drug Users, Sensitivity Analysis Using Individual-level Data for 20% Random Sample **eTable 7. **Adjusted Differential Changes in Outcomes for Established ARB vs. Comparison Drug Users, Post- vs. Pre-Recall, Sensitivity Analysis Using Individual-level Data for 20% Random Sample **eTable 8. **Differential Changes in Medication Use Outcomes, Sensitivity Analyses Using Different Cohort Inclusion Criteria **eTable 9. **Differential Changes in Drug Spending Outcomes, Sensitivity Analyses Using Different Cohort Inclusion Criteria **eTable 10. **Sensitivity Analyses Using Varying Prescription Lengths to Define Medication Gaps **eTable 11. **Adjusted Differential Changes in Outcomes, Sensitivity Analysis with Alternative Comparative Interrupted Time Series Specification Allowing for Differential Pre-Trends **eTable 12. **Descriptive Characteristics of Recalled ARB Users, by Post-Recall Switching Status **eFigure 6. **Standardized Trends in Medication Use Outcome for Recalled ARB vs. Comparison Drug Users, Post-Hoc Sensitivity Analysis by Drug **eFigure 7. **Standardized Trends in Drug Spending Outcomes for Recalled ARB vs. Comparison Drug Users, Post-Hoc Sensitivity Analysis by Drug **eTable 13. **Adjusted Differential Changes in Medication Use Outcomes for Established ARB vs. Comparison Drug Users, Post- vs. Pre-Recall, Post-Hoc Sensitivity Analysis by Drug **eTable 14. **Adjusted Differential Changes in Drug Spending Outcomes for Established ARB vs. Comparison Drug Users, Post- vs. Pre-Recall, Post-Hoc Sensitivity Analysis by Drug **eTable 15. **Reasons for Rejected Fills, Post-Hoc Sensitivity Analysis by Drug **eTable 16. **Adjusted Differential Changes in Proportion with Rx. Switch for Established ARB vs. Comparison Drug Users, Post- vs. Pre-Recall, Post-Hoc Sensitivity Analysis by Baseline MPR, Region, Prescriber Specialty, and Insurance Type

Supplement 2.
Data Sharing Statement
