# Supplementary material for: Adherence, Switches, and Drug Spending After Angiotensin Receptor Blocker Recalls and Shortages
Source: JAMA Health Forum. 2025 Nov 26;6(11):e254078. doi: 10.1001/jamahealthforum.2025.4078 (PMC12658656; doi:10.1001/jamahealthforum.2025.4078)
Supplement: Supplement 1. — eFigure 1. Definition of Study Period eTable 1. Variable Definitions eFigure 2. Cohort Selection Diagram eTable 2. Baseline Characteristics of Patients with Any ARB or Comparison Drug Fill in IQVIA Dataset, by Study Month eFigure 3. Medication Fills for Established ARB vs. Comparison Drug Users, July 2017 – December 2019, by Drug Class eFigure 4. Adjusted Event-Study Plots for Differential Changes in Medication Utilization Outcomes for Established ARB vs. Comparison Drug Users, Pre- and Post-Recall eFigure 5. Adjusted Event-Study Plots for Differential Changes in Drug Spending Outcomes for Established ARB vs. Comparison Drug Users, Pre- and Post-Recall eTable 3. Full Difference-in-Difference Models for Adjusted Changes in Medication Use Outcomes Post- vs. Pre-Recall, Established ARB vs. Comparison Drug Users eTable 4. Full Difference-in-Difference Models for Adjusted Changes in Drug Spending Outcomes Post- vs. Pre-Recall, Established ARB vs. Comparison Drug Users eTable 5. Reasons for Rejected Fills, by Period and Study Group eTable 6. Baseline Characteristics of Established ARB and Comparison Drug Users, Sensitivity Analysis Using Individual-level Data for 20% Random Sample eTable 7. Adjusted Differential Changes in Outcomes for Established ARB vs. Comparison Drug Users, Post- vs. Pre-Recall, Sensitivity Analysis Using Individual-level Data for 20% Random Sample eTable 8. Differential Changes in Medication Use Outcomes, Sensitivity Analyses Using Different Cohort Inclusion Criteria eTable 9. Differential Changes in Drug Spending Outcomes, Sensitivity Analyses Using Different Cohort Inclusion Criteria eTable 10. Sensitivity Analyses Using Varying Prescription Lengths to Define Medication Gaps eTable 11. Adjusted Differential Changes in Outcomes, Sensitivity Analysis with Alternative Comparative Interrupted Time Series Specification Allowing for Differential Pre-Trends eTable 12. Descriptive Characteristics of Recalled ARB Users, by Post-Recall Switching Status eFigu [file jamahealthforum-e254078-s001.pdf]

Supplemental Online Content

Callaway Kim K, Roberts ET, Donohue JM, et al. Adherence, switches, and drug spending after angiotensin receptor blocker recalls and shortages. *JAMA Health Forum*. 2025;6(11):e254078. doi:10.1001/jamahealthforum.2025.4078

eFigure 1. Definition of Study Period

eTable 1. Variable Definitions

eFigure 2. Cohort Selection Diagram

eTable 2. Baseline Characteristics of Patients with Any ARB or Comparison Drug Fill in IQVIA Dataset, by Study Month

eFigure 3. Medication Fills for Established ARB vs. Comparison Drug Users, July 2017 – December 2019, by Drug Class

eFigure 4. Adjusted Event-Study Plots for Differential Changes in Medication Utilization Outcomes for Established ARB vs. Comparison Drug Users, Pre- and Post-Recall

eFigure 5. Adjusted Event-Study Plots for Differential Changes in Drug Spending Outcomes for Established ARB vs. Comparison Drug Users, Pre- and Post-Recall

eTable 3. Full Difference-in-Difference Models for Adjusted Changes in Medication Use Outcomes Post- vs. Pre-Recall, Established ARB vs. Comparison Drug Users

eTable 4. Full Difference-in-Difference Models for Adjusted Changes in Drug Spending Outcomes Post- vs. Pre-Recall, Established ARB vs. Comparison Drug Users

eTable 5. Reasons for Rejected Fills, by Period and Study Group

eTable 6. Baseline Characteristics of Established ARB and Comparison Drug Users, Sensitivity Analysis Using Individual-level Data for 20% Random Sample

eTable 7. Adjusted Differential Changes in Outcomes for Established ARB vs. Comparison Drug Users, Post- vs. Pre-Recall, Sensitivity Analysis Using Individual-level Data for 20% Random Sample

eTable 8. Differential Changes in Medication Use Outcomes, Sensitivity Analyses Using Different Cohort Inclusion Criteria

eTable 9. Differential Changes in Drug Spending Outcomes, Sensitivity Analyses Using Different Cohort Inclusion Criteria

eTable 10. Sensitivity Analyses Using Varying Prescription Lengths to Define Medication Gaps

eTable 11. Adjusted Differential Changes in Outcomes, Sensitivity Analysis with Alternative Comparative Interrupted Time Series Specification Allowing for Differential Pre-Trends

© 2025 Callaway Kim K et al. *JAMA Health Forum*.

eTable 12. Descriptive Characteristics of Recalled ARB Users, by Post-Recall Switching Status

eFigure 6. Standardized Trends in Medication Use Outcome for Recalled ARB vs. Comparison Drug Users, Post-Hoc Sensitivity Analysis by Drug

eFigure 7. Standardized Trends in Drug Spending Outcomes for Recalled ARB vs. Comparison Drug Users, Post-Hoc Sensitivity Analysis by Drug

eTable 13. Adjusted Differential Changes in Medication Use Outcomes for Established ARB vs. Comparison Drug Users, Post- vs. Pre-Recall, Post-Hoc Sensitivity Analysis by Drug

eTable 14. Adjusted Differential Changes in Drug Spending Outcomes for Established ARB vs. Comparison Drug Users, Post- vs. Pre-Recall, Post-Hoc Sensitivity Analysis by Drug

eTable 15. Reasons for Rejected Fills, Post-Hoc Sensitivity Analysis by Drug

eTable 16. Adjusted Differential Changes in Proportion with Rx. Switch for Established ARB vs. Comparison Drug Users, Post- vs. Pre-Recall, Post-Hoc Sensitivity Analysis by Baseline MPR, Region, Prescriber Specialty, and Insurance Type

This supplemental material has been provided by the authors to give readers additional information about their work.

© 2025 Callaway Kim K et al. *JAMA Health Forum*.

eFigure 1: Definition of Study Period

e-Figure 1 presents a visual representation of our study period, which comprised the four quarters (1 year) before to 6 quarters (one-and-a-half years) after the first ARB recalled for valsartan in July 2018.

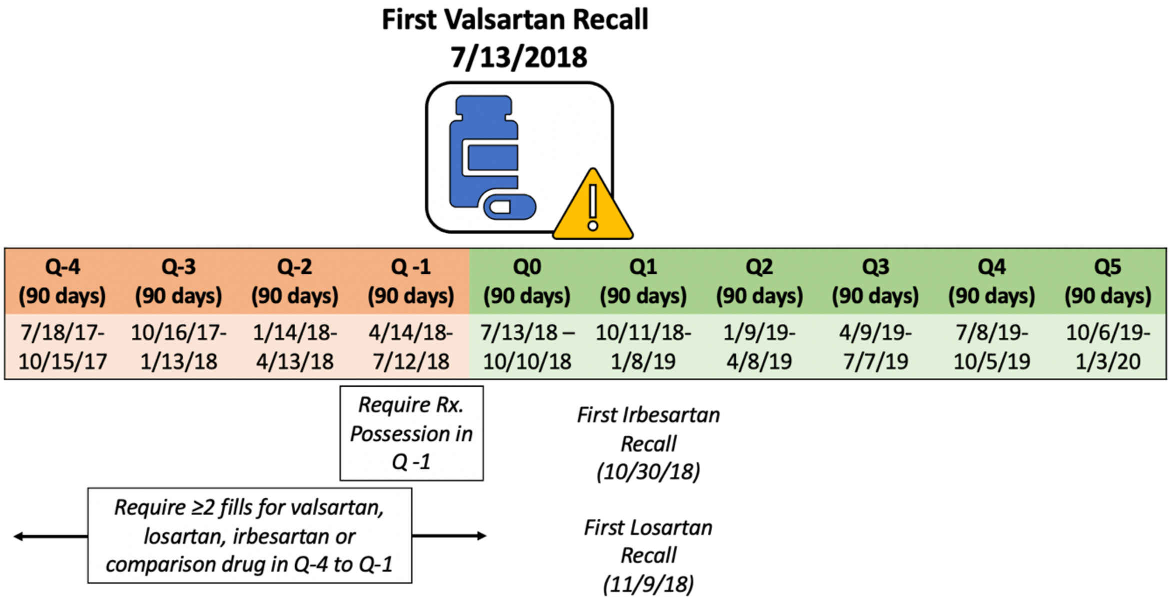

Abbreviations: ARB = angiotensin-II receptor blockers; Rx. = prescription, Q=quarter

© 2025 Callaway Kim K et al. *JAMA Health Forum*.

e-Table 1: Variable Definitions

e-Table 1 presents codes lists for the definition of study outcomes and covariates.

Variable	Definition
**Data Extract**	
Anti-hypertension drugs	Data were requested from IQVIA for all drugs belonging to the following World Health Organization Anatomical Therapeutic Chemical (ATC) classes: C09 (ARBs, ACE-Is, and other renin-angiotensin agents), C02 (antihypertensives), C03 (diuretics), C07 (beta blocking agents), C08 (calcium channel blocker). We restricted to final claims for all analyses (FINAL_CLAIM_IND= "Y").
**Study Group Assignments**	
ARB user	Recalled ARBs were defined using the "UNIFM_PROD_NM" variable in IQVIA:<br>• Valsartan: UNIFM_PROD_NM= "VALSARTAN", "VALSARTAN-HYDROCHLOROTHIAZIDE", "AMLODIPINE BESYLATE-VALSARTAN", "AMLODIPINE-VALSARTAN-HYDROCHLOROTHIAZIDE", "ALISKIREN-VALSARTAN", "NEBIVOLOL-VALSARTAN", "SACUBITRIL-VALSARTAN"<br>• Irbesartan: UNIFM_PROD_NM= "IRBESARTAN", "IRBESARTAN-HYDROCHLOROTHIAZIDE"<br>• Losartan: UNIFM_PROD_NM= "LOSARTAN POTASSIUM", "LOSARTAN POTASSIUM & HYDROCHLOROTHIAZIDE"
Comparison drug user	Comparison drugs of interest were defined using the "USC_CD" and "UNIFM_PROD_NM" variable in IQVIA:<br>• ACE-Is:<br>◦ Single agents: USC_CD=31111 or UNIFM_PROD_NM = "BENAZEPRIL HCL", "CAPTOPRIL", "ENALAPRIL MALEATE", "ENALAPRILAT", "FOSINOPRIL SODIUM", "LISINOPRIL", "LISINOPRIL-DIETARY MANAGEMENT PRODUCT", "MOEXIPRIL HCL", "PERINDOPRIL ERBUMINE", "QUINAPRIL HCL", "RAMIPRIL", "TRANDOLAPRIL"<br>◦ With hydrochlorothiazide: USC_CD=31112 or UNIFM_PROD_NM = "BENAZEPRIL & HYDROCHLOROTHIAZIDE", "CAPTOPRIL & HYDROCHLOROTHIAZIDE", "ENALAPRIL MALEATE & HYDROCHLOROTHIAZIDE", "FOSINOPRIL SODIUM & HYDROCHLOROTHIAZIDE", "LISINOPRIL & HYDROCHLOROTHIAZIDE", "MOEXIPRIL-HYDROCHLOROTHIAZIDE", "QUINAPRIL-HYDROCHLOROTHIAZIDE"<br>◦ Other combinations: USC_CD=31118 or UNIFM_PROD_NM = "AMLODIPINE BESYLATE-BENAZEPRIL HCL", "ENALAPRIL MALEATE-FELODIPINE", "PERINDOPRIL ARGININE-AMLODIPINE BESYLATE", "TRANDOLAPRIL-VERAPAMIL HCL"<br>• Non-recalled ARBs:

© 2025 Callaway Kim K et al. *JAMA Health Forum*.

- Single agents: USC_CD = 31121 or UNIFM_PROD_NM = “AZILSARTAN MEDOXOMIL”, “CANDESARTAN CILEXETIL”, “EPROSARTAN MESYLATE”, “OLMESARTAN MEDOXOMIL”, “TELMISARTAN”
- Combination products: USC_CD = 31123, USC_CD = 21124, or UNIFM_PROD_NM = “AZILSARTAN MEDOXOMIL-CHLORTHALIDONE”, “CANDESARTAN CILEXETIL-HYDROCHLOROTHIAZIDE”, “EPROSARTAN MESYLATE-HYDROCHLOROTHIAZIDE”, “OLMESARTAN MEDOXOMIL-HYDROCHLOROTHIAZIDE”, “TELMISARTAN-HYDROCHLOROTHIAZIDE”, “AMLODIPINE BESYLATE-OLMESARTAN MEDOXOMIL”, “TELMISARTAN-AMLODIPINE”, “OLMESARTAN MEDOXOMIL-AMLODIPINE-HYDROCHLOROTHIAZIDE”

Outcomes

Proportion of Days Covered (PDC)	PDC was calculated as the total number of days covered by any ARB or comparison drug supply divided by the total number of days in each quarter (90 days). PDC accounted for current and previous fills. No adjustments were made for early filling.
Rx. switch	Switches were defined as fills for different drugs which overlapped by >1 day or if a fill for a different drug occurred <30 days after an index prescription ran out. Drugs were defined using their main active ingredient so could include switches within a given drug class (e.g., lisinopril to captopril; valsartan to losartan). However, we did not include switches from a drug to combination products with the same main ingredient (e.g., valsartan to valsartan-hydrochlorothiazide did not count as a switch).
Rx. gap	Individuals were defined as experiencing a gap in medication if they had zero valsartan, losartan, irbesartan, or comparison drug supply for ≥30 days, which represented failure to fill approximately one prescription. Medication gaps were assigned to the month during which the last fill ran out. We conducted sensitivity analyses using 15, 45, 60, and 90 days to test the influence of different prescription lengths.
Mean days between fills	Days between fills were calculated as the number of days from when the last previous fill ran out to when the next fill began. We excluded patients’ last-ever fills to avoid counting discontinuations.
Payer Rx. spending	Payer spending per quarter was defined as the sum of “PRI_PAYER_PAY_AMT” variable on all ARB or comparison drug fills.
Patient OOP Rx. spending	Patient OOP spending per quarter was defined as the sum “FINAL_OPC_AMT” variable on all ARB or comparison drug fills.
Generic fills	Generic fills were defined as ARB or comparison drug fills for which the “UNIFM_PROD_NM” and “MKTED_PROD_NM” fields were equivalent (e.g., “VALSARTAN” and “VALSARTAN” versus “VALSARTAN” and “DIOVAN”.
Rejected claims	Rejected fills were defined as ARB or comparison drug fills for which the ENCNT_OUTCOME_CD variable was “RJ” (rejected).

© 2025 Callaway Kim K et al. *JAMA Health Forum*.

Reason for rejected claims	The reason for the rejected claims was determined using the RJ_CD variable on claims for which ENCT_OUTCOME_CD="RJ". Categories for rejection were:<br>• Refill restriction: RJ_CD="73" (Refills Not Covered), "79" (Refill Too Soon), "7V" (Duplicate Refills), "7W" (Refills Exceed allowable Refills), "8Q" (Excessive Refills Authorized), "9G" (Quantity Dispensed Exceeds Maximum Allowed)<br>• Step edit: RJ_CD= "608" (Step therapy, alternate drug therapy required prior to use of submitted product), "88" (DUR Reject Error)<br>• Product not covered: RJ_CD="60" (Not Covered – Age), "61" (Not Covered – Gender), "63" (Institutionalized Patient Product/Service ID Not Covered), "65" (Patient Not Covered), "70" (Not Covered), "7Y" (Compounds Not Covered), "8A" (Compound Requires At Least One Covered Ingredient), "8H" (Product/Service Only Covered On Compound Claim), "9Q" (Route Of Administration Submitted Not Covered), "9R" (Prescription/Service Reference Number Qualifier Submitted Not Covered), "9T" (Prior Authorization Type Code Submitted Not Covered), "9Y" (Compound Product ID Qualifier Submitted Not Covered), "BB" (Diagnosis Code Qualifier Submitted Not Covered), "MR"(Drug Not on Formulary)<br>• Plan limitations exceeded: RJ_CD= "76" (Plan Limits Exceeded), "7X"(Days Supply Exceeds Plan Limitation), "AG" (Days Supply Limit), "RN" (Plan Limits Exceeded On Intended Partial Fill Transaction)<br>• Prior authorization required: RJ_CD= "3N" (M/I Prior Authorized Number Assigned), "3P" (M/I Authorization Number), "3S" (M/I Prior Authorization Supporting Documentation), "3T" (Active Prior Authorization Exists Resubmit At Expiration Of Prior Authorization), "3W" (Prior Authorization In Process), "3X" (Authorization Number Not Found), "3Y" (Prior Authorization Denied), "64" (Claim Submitted Does Not Match Prior Authorization), "6Q" (Prior Authorization Segment Required For Adjudication), "75" (Prior Auth Required), "80" (Drug-Diagnosis Mismatch), "EU" (M/I Prior Authorization Type Code), "EV" (M/I Prior Authorization Number Submitted), "MV" (M/I Benefit Stage Qualifier), "PA" (PA Exhausted/Not Renewable)<br>• Distribution limitation: RJ_CD="40" (Pharmacy Not Contracted With Plan On Date Of Service), "4W" (Must Fill Through Specialty Pharmacy), "50" (Non-Matched Pharmacy Number), "R6" (Product/Service Not Appropriate For This Location)<br>• Missing or invalid information on claim: All other RJ_CDs not listed above.

Covariates

Patient age and gender	Patient age and gender were defined using the IQVIA variables on the most recent pre-recall fill. Age was categorized into the following categories: 18-20 years, 20-29 years, 30-39 years, 40-49 years, 50-63 years, 65-79 years, and ≥80 years. We excluded individuals with unknown or missing gender.
Region and residential location based on 3-level ZIP code	Individuals were assigned to a single region and 3-level ZIP code based on the most fills in the pre-recall period (7/17/2017-7/12/2018). We then merged the 3-level ZIP codes to 5-level ZIP codes in the 2010 Rural Urban Commuting Area (RUCA) ZIP code files. Using 2010 Census data and Heath Resources and Services Administration (HRSA) definitions, 3-level ZIP codes for which ≥50% of the population lived in 5-level ZIP codes "1" (Metropolitan area core: primary flow within an urbanized area), "2"

© 2025 Callaway Kim K et al. *JAMA Health Forum*.

(Metropolitan area high commuting: primary flow 30% or more to an urbanized area) or “3” (Metropolitan area low commuting: primary flow 10% to 30% to an urbanized area) were classified as metropolitan. ZIP codes with <50% of the population in “1”, “2”, or “3” RUCA areas were classified as non-metropolitan.

Most common fill location pre-recall	Individuals were assigned to a single predominant fill location based on the most fills in the pre-recall period and the “CHNL_CD” variable in IQVIA. If ≥75% of fills had CHNL_CD= “R” then patients were categorized as predominantly retail pharmacy users. The same 75% cut-off was used to identify predominantly long-term-care (CHNL_CD= “L”) and predominantly mail-order (CHNL_CD= “M”) users. Individuals with <75% of fills from a single location were categorized into the unknown/mixed/missing group.
Insurance type on last pre-recall fill	Using the “PAY_TYP_CD” variable in the FIA claims, individuals were assigned to a single predominant insurance type based on their most recent pre-recall fill. Categories included: Medicare (PAY_TYPE_CD = 4 Medicare or PAY_TYPE_CD=5 Medicare Part D), Third Party (PAY_TYPE_CD=3), Medicaid fee-for-service (PAY_TYPE_CD = 2), or Cash (PAY_TYPE_CD=1). We excluded fills with missing/unknown payer. Per IQVIA, the Third-Party category included coverage from an employer, Medicaid Managed Care, and individual market plans. We did not have access to reliable variables to distinguish between these types.
Primary prescriber specialty pre-recall	Individuals were assigned to a single primary prescriber (NPI) based on the most fills in the pre-recall period (7/17/2017-7/12/2018). To define prescriber specialty, we used the “PRFN_TYP_ID” and “PROVIDER_PRI_SPCL_CD” variables in IQVIA:<br><br>We first identified advanced practice providers as those with a PRFN_TYPE_ID = “7” Physicians Assistants or with a PROVIDER_PRI_SPCL_CD = NRP (nurse practitioner)<br><br>Among medical doctors (PRFN_TYP_IDs “1” Medical Doctors or “2” Doctors of Osteopathy), we defined primary specialty using the PROVIDER_PRI_SPCL_CD variable:<br>• Internal medicine or other general practice: PROVIDER_PRI_SPCL_CDs = FM (family medicine), GP (general practice), GPM (general preventive medicine), HOS (hospitalist), IFP (internal medicine/family medicine), IM (internal medicine), IPM (internal medicine/preventive medicine), or PHP (public health & general preventive medicine)<br>• Cardiovascular disease: PROVIDER_PRI_SPCL_CDs = AHF (advanced heart failure & transplant cardiology), CD (cardiovascular disease), CHD (adult congenital heart disease (internal medicine)), IC (interventional cardiology), NC (nuclear cardiology), or PDC (pediatric cardiology)<br>• Nephrology: PROVIDER_PRI_SPCL_CDs = NEP (nephrology) or PN (pediatric nephrology)<br>• Diabetes, endocrine, or other metabolic disease: PROVIDER_PRI_SPCL_CDs = DIA (diabetes), END (endocrinology, diabetes & metabolism), PDE (pediatric endocrinology), REN (reproductive endocrinology & infertility), RGM (reproductive endocrinology & infertility/mgm)<br>• All other specialties were grouped together.

© 2025 Callaway Kim K et al. *JAMA Health Forum*.

Pre-recall use of other anti-hypertension drug classes	Other anti-hypertension drug classes were defined using the “USC_CD” variable in IQVIA:<br>• Calcium channel blockers: USC_CD=31330 or UNIFM_PROD_NM = “AMLODIPINE & DIETARY MANAGEMENT PRODUCT”, “AMLODIPINE BENZOATE”, “AMLODIPINE BESYLATE”, “BEPRIDIL HCL”, “CLEVIDIPINE”, “DILTIAZEM HCL”, “DILTIAZEM HCL COATED BEADS”, “DILTIAZEM HCL EXTENDED RELEASE BEADS”, “DILTIAZEM HCL IN DEXTROSE”, “DILTIAZEM HCL IN SODIUM CHLORIDE”, “DILTIAZEM MALATE”, “FELODIPINE”, “ISRADIPINE”, “LEVAMLODIPINE MALEATE”, “MIBEFRADIL DIHYDROCHLORIDE”, “NICARDIPINE HCL”, “NICARDIPINE HCL IN DEXTROSE”, “NICARDIPINE HCL IN SODIUM CHLORIDE”, “NIFEDIPINE”, “NIMODIPINE”, “NISOLDIPINE”, “VERAPAMIL HCL”<br>• Diuretics: USC_CD=41110, 41120, 41130, 41140 or 41190 or UNIFM_PROD_NM= “BENDROFLUMETHIAZIDE”, “CHLOROTHIAZIDE”, “CHLOROTHIAZIDE SODIUM”, “CHLORTHALIDONE”, “HYDROCHLOROTHIAZIDE”, “INDAPAMIDE”, “METHYCLOTHIAZIDE”, “METOLAZONE”, “POLYTHIAZIDE”, “TRICHLORMETHIAZIDE”, “BUMETANIDE”, “ETHACRYNATE SODIUM”, “ETHACRYNIC ACID”, “FUROSEMIDE”, “FUROSEMIDE IN SODIUM CHLORIDE”, “TORSEMIDE”, “AMILORIDE HCL”, “SPIRONOLACTONE”, “TRIAMTERENE”, “AMILORIDE & HYDROCHLOROTHIAZIDE”, “BUCHU-ALF-ASPARAGUS-K GLUC-COUCH GR-PARSLEY-UVA URSI-JUNIPER”, “BUCHU-CAF-MG TRIS-M BLUE-K SAL-SALUVA URSI-JUNIPER”, “BUCHU-CORNSILK-COUCH GRASSHYDRANGEA”, “BUCHU-JUNIPER-K GLUC-PARSLEY-UVA URSI”, “CAFFEINE-MAGNESIUM SALICYLATE”, “RESERPINE & HYDROCHLOROTHIAZIDE”, “SPIRONOLACTONE & HYDROCHLOROTHIAZIDE”, “TRIAMTERENE & HYDROCHLOROTHIAZIDE”, “ACETAMINOPHEN W/ PAMABROM”, “AMMONIUM CHLORIDE”, “GLYCERIN”, “PAMABROM”<br>• Adrenergic blockers: USC_CD = 31410, 31420, 31430, 31440, or 31450, or UNIFM_PROD_NM= ”ACEBUTOLOL HCL”, “ATENOLOL”, “BETAXOLOL HCL”, “BISOPROLOL FUMARATE”, “ESMOLOL HCL”, “ESMOLOL HCL-SODIUM CHLORIDE”, “METOPROLOL SUCCINATE”, “METOPROLOL TARTRATE”, “METOPROLOL TARTRATE-DIETARY MANAGEMENT PRODUCT”, “NADOLOL”, “NEBIVOLOL HCL”, “PENBUTOLOL SULFATE”, “PINDOLOL”, “PROPRANOLOL HCL”, “PROPRANOLOL HCL SUSTAINED-RELEASE BEADS”, “TIMOLOL MALEATE”, “CARVEDILOL”, “CARVEDILOL PHOSPHATE”, “LABETALOL HCL”, “LABETALOL HCL-DEXTROSE”, “LABETALOL HCL-SODIUM CHLORIDE”, “ATENOLOL & CHLORTHALIDONE”, “BISOPROLOL & HYDROCHLOROTHIAZIDE”, “METOPROLOL & HYDROCHLOROTHIAZIDE”, “NADOLOL & BENDROFLUMETHIAZIDE”, “PROPRANOLOL & HYDROCHLOROTHIAZIDE”, “DOXAZOSIN MESYLATE”, “DOXAZOSIN MESYLATE (BPH)”, “PHENOXYBENZAMINE HCL”, “PRAZOSIN & POLYTHIAZIDE”, “PRAZOSIN HCL”, “TERAZOSIN HCL”, “CLONIDINE”, “CLONIDINE & CHLORTHALIDONE”, “CLONIDINE HCL”, “GUANABENZ ACETATE”, “GUANFACINE HCL”, “METHYLDOPA”, “METHYLDOPA & CHLOROTHIAZIDE”, “METHYLDOPA & HYDROCHLOROTHIAZIDE”, “METHYLDOPATE HCL”<br>• Other vascular agents: USC_CD=31900 or UNIFM_PROD_NM= “AMLODIPINE BESYLATE-CELECOXIB”, “DESERPIDINE & METHYCLOTHIAZIDE”, “FENOLDOPAM MESYLATE”, “GUANADREL SULFATE”, “GUANETHIDINE MONOSULFATE”, “HYDRALAZINE & HYDROCHLOROTHIAZIDE”, “HYDRALAZINE & RESERPINE & HYDROCHLOROTHIAZIDE”, “HYDRALAZINE HCL”, “MECAMYLAMINE HCL”, “METYROSINE”, “MINOXIDIL”, “NITROPRUSSIDE SODIUM”, “NITROPRUSSIDE SODIUM-

© 2025 Callaway Kim K et al. *JAMA Health Forum*.

SODIUM CHLORIDE”, “PHENTOLAMINE MESYLATE”, “RESERPINE”, “RESERPINE & CHLOROTHIAZIDE”, “RESERPINE & HYDROCHLOROTHIAZIDE”, “RESERPINE & HYDROFLUMETHIAZIDE”, “RESERPINE & METHYCLOTHIAZIDE”

- Other renin-acting agents: USC_CD=31130, 31142, or 31149, or UNIFM_PROD_NM= “EPLERONE”, “FINERENOME”, “ALISKIREN FUMARATE”, “ALISKIREN-HYDOCHLOROTHIAZIDE”, “ALISKIREN-AMLODIPINE”, “ALISKIREN-AMPLODIPINE-HYDOCHLOROTHIAZIDE”

Abbreviations: ARB= angiotensin-receptor-II-blocker; PDC = proportion of days covered; Rx. = prescription; OOP = out-of-pocket; NPI=National Provider Identifier

© 2025 Callaway Kim K et al. *JAMA Health Forum*.

e-Figure 2: Cohort Selection Diagram

e-Figure 2 presents a flow diagram of our inclusion and exclusion criteria.

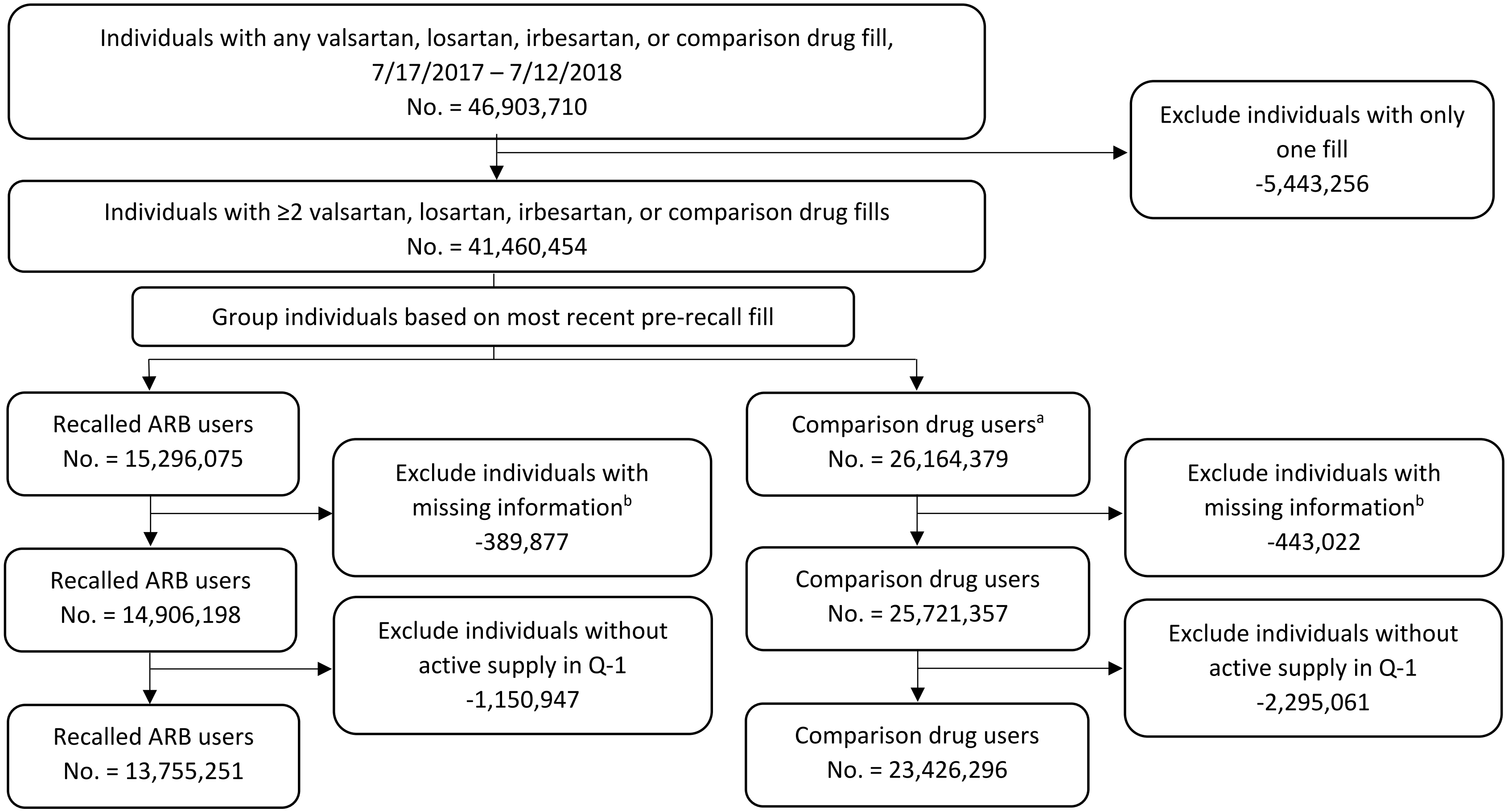

Abbreviations: ARB = angiotensin-receptor-II blocker; No. = number

a. Comparison drugs included ACE-Is and non-recalled ARBs (e-Table 1).

b. Individuals were excluded if they had a primary residence in Puerto Rico (353,759 ARB and 249,368 comparison drug users), were <18 years old in 2018 (32,216 ARB and 183,486 comparison drug users), had missing or unknown gender (3,474 ARB and 9,198 comparison drug users), or had missing or unknown payer type for their last pre-recall fill (428 ARB and 970 comparison drug users).

© 2025 Callaway Kim K et al. *JAMA Health Forum*.

e-Table 2: Baseline Characteristics of Individuals with Any ARB or Comparison Drug Fill in IQVIA Dataset, by Study Month

e-Table 2 presents descriptive characteristics of individuals in the IQVIA FIA dataset with any anti-hypertension drug fill, by study period.

Variable	12 months pre-valsartan-recall (July 2017)	1 month pre-valsartan recall (June 2018)	First Irbesartan and Losartan Recalls (October 2018)	12 months post-valsartan-recall (July 2019)	18 months post-valsartan-recall (December 2019)
Study Group					
Users of Recalled ARBs at Baseline	6,150,726	6,744,397	6,436,028	5,100,135	4,977,772
Users of Comparison Drugs at Baseline	10,326,092	11,368,362	10,542,175	8,625,158	8,414,651
Median (IQR) age in 2018	63 (55,72)	62 (54,71)	63 (54,72)	62 (54,71)	62 (54,71)
Age group in 2018					
18-20	14,176 (0.1)	18,022 (0.1)	14,678 (0.1)	10,778 (0.1)	9,580 (0.1)
20-29	108,543 (0.7)	156,129 (0.9)	127,354 (0.8)	95,163 (0.7)	88,545 (0.7)
30-39	530,143 (3.3)	709,949 (4.0)	607,462 (3.7)	474,546 (3.5)	450,075 (3.4)
40-49	1,751,313 (11)	2,130,031 (12.1)	1,915,066 (11.7)	1,556,609 (11.7)	1,507,027 (11.3)
50-64	6,552,456 (39.7)	7,329,854 (40.5)	6,865,259 (40.4)	5,665,168 (41.3)	5,521,568 (41.2)
65-79	5,662,085 (34.4)	5,867,617 (32.4)	5,651,394 (33.3)	4,556,024 (33.2)	4,521,334 (33.8)
≥80	1,858,102 (11.3)	1,901,157 (10.5)	1,796,990 (10.6)	1,367,005 (10.0)	1,294,294 (9.7)
Gender					
Female	8,139,577 (49.4)	8,915,411 (49.2)	8,363,087 (49.3)	6,729,377 (49.0)	6,532,381 (48.8)
Male	8,337,241 (50.6)	9,197,348 (50.8)	8.615,116 (50.7)	6,995,916 (51.0)	6,860,042 (51.2)
U.S. region					
Northeast	2,833,618 (17.2)	3,128,608 (17.3)	2,950,751 (17.4)	2,362,100 (17.2)	2,286,845 (17.1)
Midwest	3,552,083 (21.6)	3,905,600 (21.6)	3,696,066 (21.8)	3,037,231 (22.1)	3,005,428 (22.4)
South	6,771,255 (41.1)	7,343,398 (40.5)	6,831,856 (40.2)	5,519,448 (40.2)	5,376,427 (40.1)
West	2,888,995 (17.5)	3,265,155 (18.0)	3,038,812 (17.9)	2,445,120 (17.8)	2,363,637 (17.6)
Missing/unknown	430,867 (2.6)	469,998 (2.6)	460,718 (2.7)	361,394 (2.6)	360,086 (2.7)
Patient residential location based on 3-level ZIP code					
<50% metropolitan by population	1,960,477 (11.9)	2,136,474 (11.8)	2,007,117 (11.8)	1,624,346 (11.8)	1,599,001 (11.9)
≥50% metropolitan by population[b]	13,013,776 (79.0)	14,314,817 (79.0)	13,402,830 (78.9)	10,849,427 (79.0)	10,573,701 (79)

© 2025 Callaway Kim K et al. *JAMA Health Forum*.

Missing/unknown	1,502,565 (9.1)	1,661,468 (9.2)	1,68,256 (9.2)	1,251,520 (9.1)	1,219,721 (9.1)
Median (IQR) pre-recall PDC	93.9 (84.5,97.7)	90.8 (76.4,96.8)	91.7 (78.4,97.1)	92.0 (79.0,97.1)	92.0 (79.3,97.4)
Pre-recall PDC category					
Low (<50 p.p.)	320,783 (1.9)	61,926 (4.8)	654,028 (3.9)	507,263 (3.7)	482,928 (3.6)
Moderate (51-79 p.p.)	2,803,253 (17.0)	4,483,573 (24.8)	3,909,829 (23.0)	3,100,284 (22.6)	2,981,196 (22.3)
High (≥80 p.p.)	13,352,782 (81.0)	12,767,260 (70.5)	12,414,346 (73.1)	10,117,746 (73.7)	9,928,299 (74.1)
Most common fill location pre-recall					
Retail pharmacy	14,606,626 (88.6)	15,994,687 (88.3)	14,943,477 (88.0)	12,081,643 (88.0)	11,720,013 (88)
Mail-order pharmacy	1,173,527 (7.1)	1,266,694 (7.0)	1,265,523 (7.5)	1,039,017 (7.6)	1,083,072 (8.1)
Long term care facility	157,951 (1.0)	204,986 (1.1)	177,103 (1.0)	130,150 (0.9)	117,944 (0.9)
Unknown/mixed/missing	538,714 (3.3)	646,392 (3.6)	592,100 (3.5)	474,483 (3.5)	471,394 (3.5)
Insurance type on last pre-recall fill					
Medicare[c]	6,296,004 (38.2)	6,437,175 (35.5)	6,179,269 (36.4)	4,910,329 (35.8)	4,831,875 (36.1)
Third-Party[d]	9,528,330 (57.8)	10,881,232 (60.1)	10,110,971 (59.6)	8,258,676 (60.2)	8,028,447 (59.9)
Medicaid fee-for-service	246,213 (1.5)	312,289 (1.7)	276,104 (1.6)	220,320 (1.6)	207,357 (1.5)
Cash/self-pay	406,271 (2.5)	482,063 (2.7)	411,859 (2.4)	335,968 (2.4)	324,744 (2.4)
Primary prescriber specialty pre-recall[e]					
Internal medicine or other general practice	10,815,750 (65.6)	11,689,010 (64.5)	11,067,844 (65.2)	9,005,935 (65.6)	8820,895 (65.9)
Physician's assistant or nurse practitioner	2,991,008 (18.2)	3,471,403 (19.2)	3,162,341 (18.6)	2,541,932 (18.5)	2457,625 (18.4)
Cardiovascular disease	1,527,762 (9.3)	159,290 (9.2)	1,576,904 (9.3)	1,252,096 (9.1)	1,222,162 (9.1)
Nephrology	237,043 (1.4)	263,260 (1.5)	238,794 (1.4)	183,432 (1.3)	174,087 (1.3)
Diabetes, endocrine, or other metabolic disease	176,560 (1.1)	190,325 (1.0)	180,075 (1.1)	147,963 (1.1)	16,754 (1.1)
Other specialty	600,736 (3.6)	685,502 (3.8)	623,845 (3.7)	493,995 (3.6)	475,693 (3.6)
Missing/unknown	127,959 (0.8)	153,969 (0.9)	128,400 (0.8)	99,940 (0.7)	95,207 (0.7)
Pre-recall anti-hypertension medication use category					
1 drug class	6,554,023 (39.8)	7,367,537 (40.7)	6,900,441 (40.6)	5,641,533 (41.1)	5,539,323 (41.4)
2 drug classes	5,507,765 (33.4)	6,021,024 (33.2)	5,678,276 (33.4)	4,601,223 (33.5)	4,494,837 (33.6)
3 drug classes	3,217,498 (19.5)	3,454,878 (19.1)	3,230,792 (19.0)	2,572,125 (18.7)	2,487,254 (18.6)
≥4 drug classes	1,197,532 (7.3)	1,269,320 (7.0)	1,168,694 (6.9)	910,412 (6.6)	871,009 (6.5)

© 2025 Callaway Kim K et al. *JAMA Health Forum*.

Pre-recall use of other anti-hypertension drug classes[f]					
Calcium channel blockers	4,607,209 (28.0)	4,948,525 (27.3)	4,641,387 (27.4)	3,718,037 (27.1)	3,607,603 (26.9)
Diuretics	4,575,618 (27.8)	4,965,586 (27.4)	4,611,743 (27.2)	3,668,065 (26.7)	3,543,212 (26.5)
Adrenergic blockers	6,077,969 (36.9)	6530,639 (36.1)	6,124,108 (36.1)	4,883,735 (35.6)	47,35,514 (35.4)
Other renin-angiotensin agents	28,162 (0.2)	30,424 (0.2)	28,912 (0.2)	23,163 (0.2)	22,657 (0.2)
Other vascular agents	379,236 (2.3)	404,682 (2.2)	365,410 (2.2)	277,544 (2.0)	261,394 (2.0)

Abbreviations: ARB = angiotensin-II-receptor blocker; Std. Diff. = standardized difference; PDC= proportion of days covered; p.p. = percentage points; IQR = interquartile range

a. Non-recalled ARBs included azilsartan, candesartan, eprosartan, olmesartan, and telmisartan, including combination products (see e-Table 1 in Supplement).
b. Areas were defined as predominantly metropolitan if ≥50% of the population resided in ZIP codes classified as 2010 Rural Urban Commuting Areas “1” (Metropolitan area core), “2” (Metropolitan area high commuting) or “3” (Metropolitan area low commuting).
c. Medicare insurance type included both Medicare and Medicare Part D.
d. Third party insurance included coverage from an employer, Medicaid Managed Care, and individual market plans. We did not have access to reliable variables to distinguish between these types.
e. Patients were assigned a single primary prescriber based on the most fills in the pre-recall period (7/18/2017 – 7/12/2018). See e-Table 1 in Supplement for codes used to define each prescriber specialty.
f. Specific drugs included in each anti-hypertension class can be found in e-Table 1 in the Supplement.

© 2025 Callaway Kim K et al. *JAMA Health Forum*.

e-Figure 3: Medication Fills for Established ARB vs. Comparison Drug Users, July 2017 – December 2019, by Drug Class[a]

A. Users of Recalled ARBs at Baseline

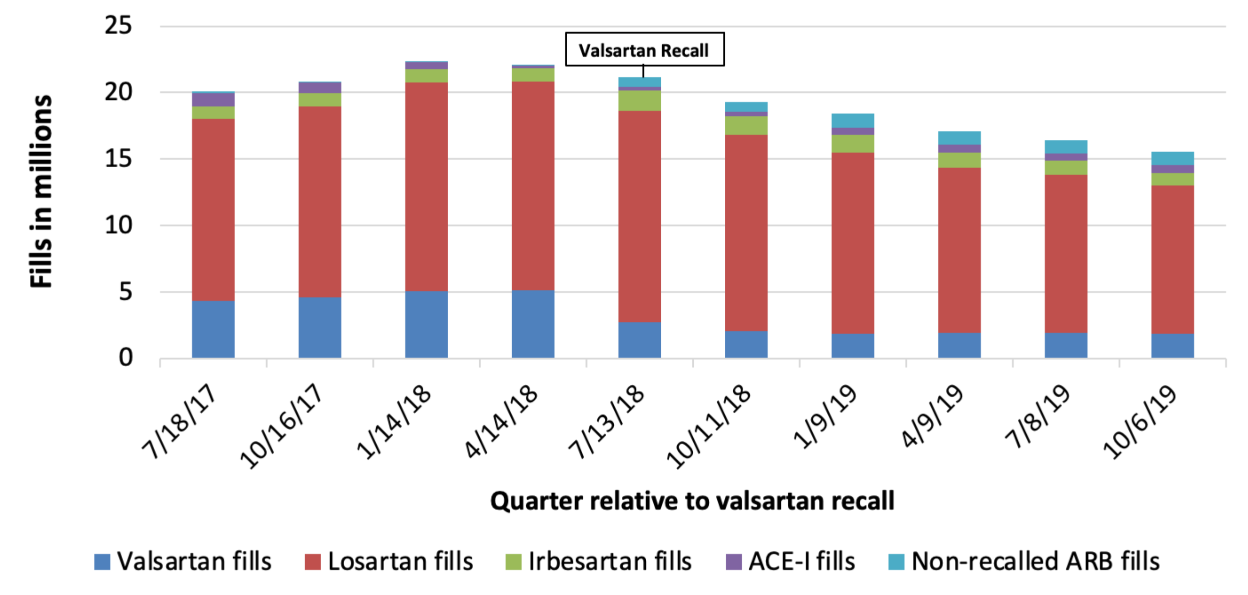

B. Users of Comparison Drugs at Baseline

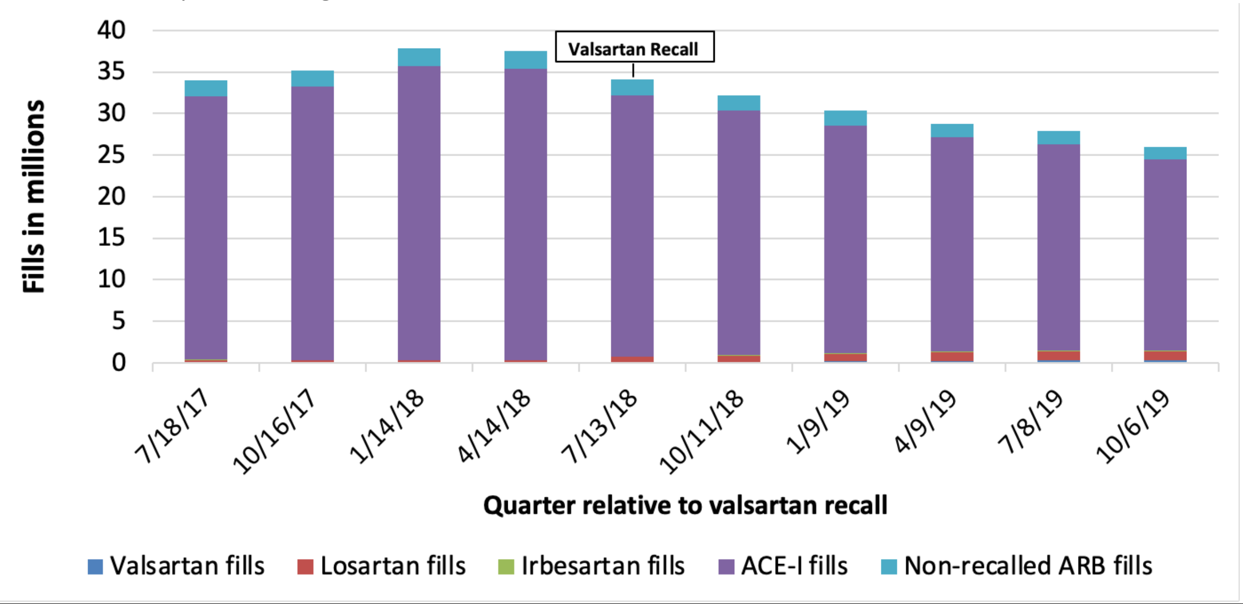

Abbreviations: ARB = angiotensin-receptor-II blocker; ACE-I = angiotensin-converting-enzyme inhibitor

a. Quarters were defined as 90-day intervals indexed to the first ARB recall for valsartan on July 13, 2018. Established users were defined as those individuals with ≥2 fills in the four quarters pre-valsartan recall (Q-4 to Q-1) and medication supply in the 90 days immediately pre-recall (Q-1). Individuals were censored for months during which they had zero days' supply for any ARB or any ACE-I. The number (proportion) with any supply was: 11,525,076 (86.3%) in Q-4, 13,352,284 (100%) in Q-1; 12,028,369 (90.1%) in Q2, and 11,016,154 (82.5%) in Q4 for ARB users and 19,475,825 (85.7%) in Q-4, 22,722,215 (100%) in Q-1, 20,039,146 (92.6%) in Q2 and 18,628,909 (82.0%) in Q4 for comparison drug users, respectively.

© 2025 Callaway Kim K et al. *JAMA Health Forum*.

e-Figure 4: Adjusted Event-Study Plots for Differential Changes in Medication Utilization Outcomes for Established ARB vs. Comparison Drug Users, Pre- and Post-Recall[a]

e-Figure 4 presents adjusted event-study difference-in-difference estimates comparing differential changes in medication use outcomes post- vs. pre-recall for ARB vs. comparison drug users.

A. Mean PDC

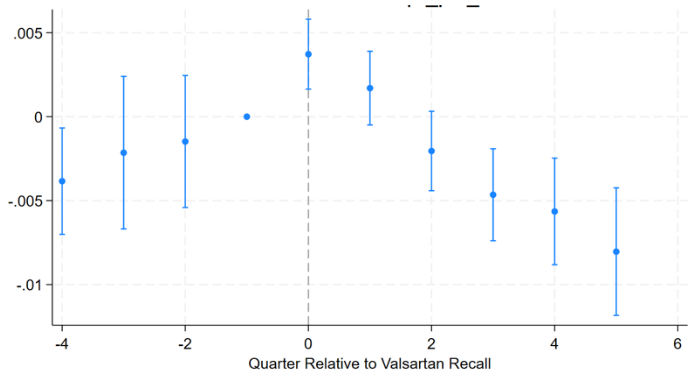

Test for pre-trend[b] p-value = 0.1155

B. Proportion with Rx. Switch

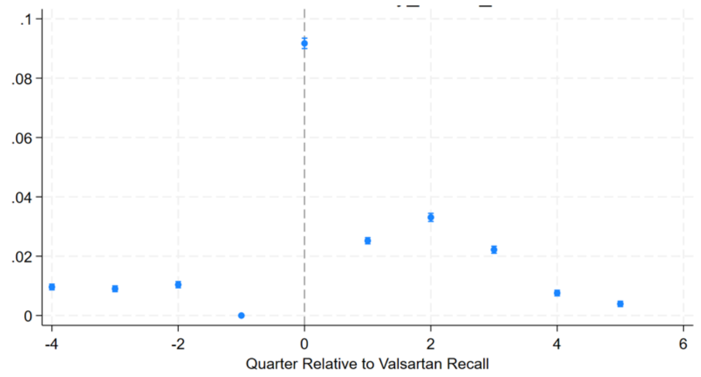

Test for pre-trend[b] p-value < 0.001

C. Proportion with Rx. Gap ≥30 days

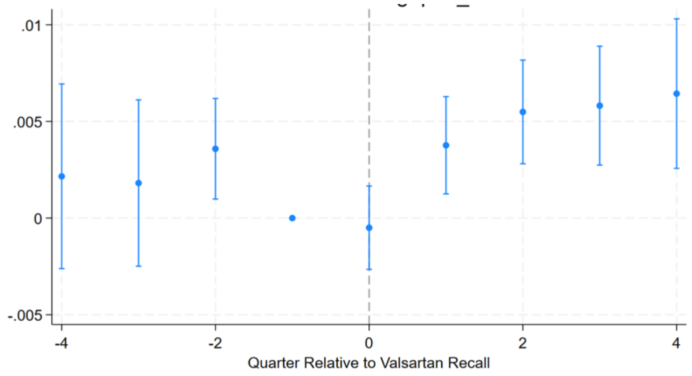

Test for pre-trend[b] p-value = 0.0594

D. Mean Days Between Fills

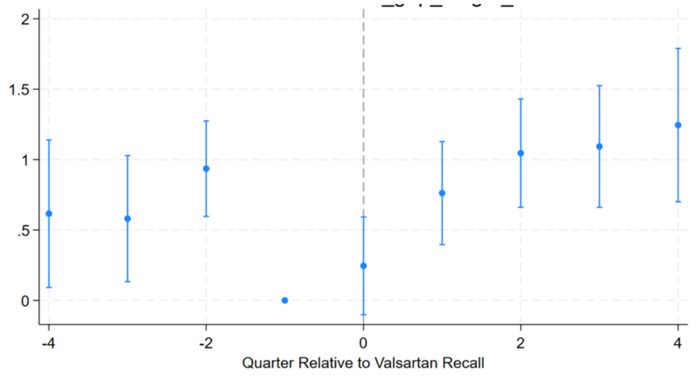

Test for pre-trend[b] p-value < 0.001

<u>Abbreviations</u>: PDC = proportion of days covered; Rx. = prescription

a. Quarters were defined as 90-day intervals indexed to the first ARB recall for valsartan on July 13, 2018. Q-1 (4/14/2018 – 7/12/2018) was used as the reference quarter. Estimates are from difference-in-difference models comparing differential changes in outcomes for ARB versus comparison drug users in each quarter. Models were adjusted for age group (19-20, 20-29, 30-39, 40-49, 50-64, 65-79, ≥80 years), female gender, pre-recall fill location (retail, long-term-care, mail-order, or unknown/mixed), pre-recall anti-hypertension medication use category (1, 2, 3, or ≥4 drug classes), pre-recall PDC category (<50, 51-79, ≥80 p.p.), primary prescriber specialty, U.S. geographic region, and patient metropolitan location.
b. Global test for the null hypothesis that all event-study estimates prior to 7/13/2018 were jointly equal to zero. Rejection of the null hypothesis (p-value < 0.05) suggests potential violation of the parallel pre-trend assumption.

© 2025 Callaway Kim K et al. *JAMA Health Forum*.

e-Figure 5: Adjusted Event-Study Plots for Differential Changes in Drug Spending Outcomes for Established ARB vs. Comparison Drug Users, Pre- and Post-Recall[a]

e-Figure 5 presents adjusted event-study difference-in-difference estimates comparing differential changes in drug spending outcomes post- vs. pre-recall for ARB vs. comparison drug users.

A. Mean Insurer Spending per Patient

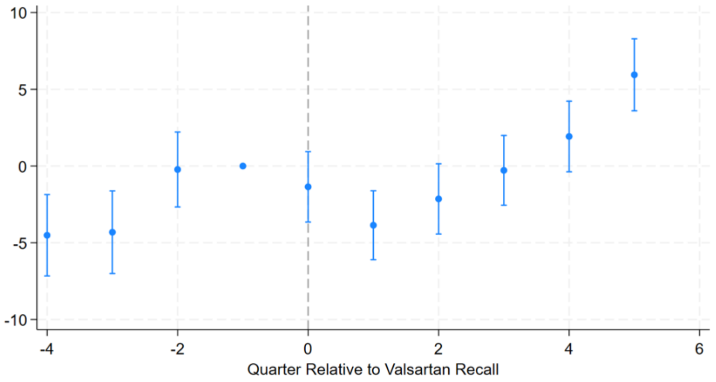

Test for pre-trend[b] p-value = 0.0002

B. Mean OOP Spending per Patient

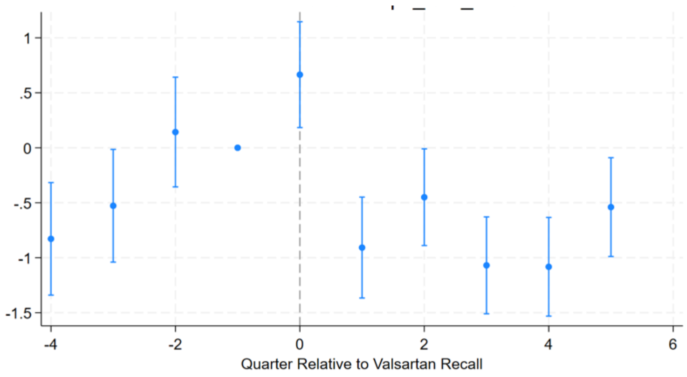

Test for pre-trend[b] p-value = 0.0006

C. Generic Fills per Patient

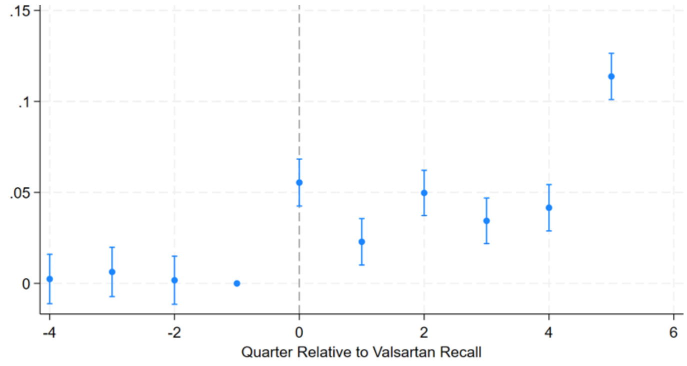

Test for pre-trend[b] p-value = 0.8275

D. Rejected Fills per Patient

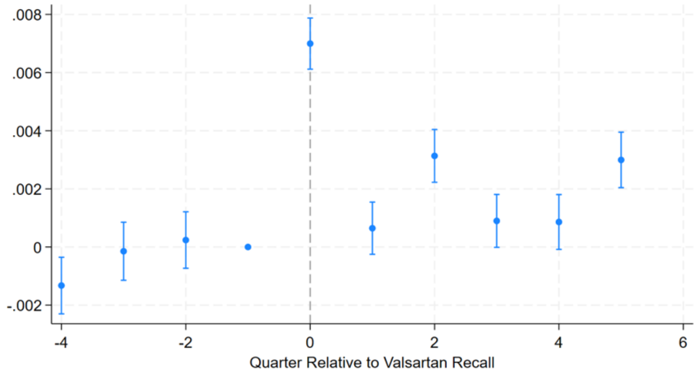

Test for pre-trend[b] p-value = 0.0036

Abbreviations: OOP = out-of-pocket

a. Quarters were defined as 90-day intervals indexed to the first ARB recall for valsartan on July 13, 2018. Q-1 (4/14/2018 – 7/12/2018) was used as the reference quarter. Estimates are from difference-in-difference models comparing differential changes in outcomes for ARB versus comparison drug users in each quarter, relative to one quarter pre-valsartan recall. Models were adjusted for age group (19-20, 20-29, 30-39, 40-49, 50-64, 65-79, ≥80 years), female gender, pre-recall fill location (retail, long-term-care, mail-order, or unknown/mixed), pre-recall anti-hypertension medication use category (1, 2, 3, or ≥4 drug classes), pre-recall PDC category (<50, 51-79, ≥80 p.p.), primary prescriber specialty, U.S. geographic region, and patient metropolitan residential location.

b. Global test for the null hypothesis that all event-study estimates prior to 7/13/2018 were jointly equal to zero. Rejection of the null hypothesis (p-value < 0.05) suggests potential violation of the parallel pre-trend assumption.

© 2025 Callaway Kim K et al. *JAMA Health Forum*.

e-Table 3: Full Difference-in-Difference Models for Adjusted Changes in Medication Use Outcomes Post- vs. Pre-Recall, Established ARB vs. Comparison Drug Users

e-Table 3 presents the full output from our main difference-in-difference models for medication use outcomes.

Variable	Mean Proportion of Days Covered		Proportion with Rx. Switch per Quarter		Proportion with Rx. Gap ≥30 days per Quarter		Mean Days Between Fills	
	Estimate (95% CI)	p-value	Estimate (95% CI)	p-value	Estimate (95% CI)	p-value	Estimate (95% CI)	p-value
Intercept	45.43 (45.09,45.76)	<0.001	-0.76 (-0.84, -0.68)	<0.001	34.74 (34.38,35.09)	<0.001	37.8 (37.38,38.23)	<0.001
ARB vs. comparison drug user at baseline	0.19 (0.03,0.35)	0.021	1.87 (1.83,1.90)	<0.001	-0.32 (-0.48, -0.16)	<0.001	-0.40 (-0.55, -0.24)	<0.001
After Valsartan Recall (7/13/18 – 10/10/18)	-0.35 (-0.49, -0.21)	<0.001	0.66 (0.62,0.70)	<0.001	0.31 (0.16,0.46)	<0.001	1.71 (1.58,1.85)	<0.001
ARB user *Post-Valsartan-Recall	0.55 (0.35,0.76)	<0.001	8.46 (8.30,8.63)	<0.001	-0.24 (-0.45, -0.03)	0.028	-0.28 (-0.46, -0.10)	0.003
After Losartan and Irbesartan Recalls (10/11/18 – 1/3/20)	-0.79 (-0.93, -0.65)	<0.001	0.70 (0.68,0.73)	<0.001	-1.60 (-1.75, -1.45)	<0.001	-1.84 (-1.99, -1.68)	<0.001
ARB user*Post-Losartan-Irbesartan-Recalls	-0.17 (-0.36,0.02)	0.087	1.20 (1.12,1.27)	<0.001	0.35 (0.14,0.56)	0.001	0.51 (0.29,0.72)	<0.001
Female gender	-0.21 (-0.30, -0.12)	<0.001	0.07 (0.04,0.10)	<0.001	0.15 (0.05,0.25)	0.003	0.11 (0.01,0.21)	0.0028
Age group in 2018								
<20	Ref.	Ref.	Ref.	Ref.	Ref.	Ref.	Ref.	Ref.
20-29	-0.31 (-0.52, -0.1)	0.003	-0.13 (-0.2, -0.07)	<0.001	-0.39 (-0.61, -0.17)	0.001	0.40 (0.11,0.68)	0.006
30-39	0.73 (0.52,0.94)	<0.001	-0.13 (-0.2, -0.06)	<0.001	-0.31 (-0.53, -0.08)	0.007	0.25 (-0.03,0.53)	0.076
40-49	2.18 (1.99,2.37)	<0.001	-0.05 (-0.11,0.02)	0.175	-0.33 (-0.55, -0.12)	0.002	-0.25 (-0.51,0)	0.049
50-64	4.02 (3.85,4.20)	<0.001	0.05 (-0.01,0.12)	0.102	-1.81 (-2.01, -1.62)	<0.001	-2.26 (-2.49, -2.03)	<0.001
65-79	5.1 (4.94,5.27)	<0.001	0.00 (-0.06,0.07)	0.941	-1.52 (-1.71, -1.33)	<0.001	-2.13 (-2.35, -1.91)	<0.001
≥80	4.55 (4.38,4.73)	<0.001	-0.28 (-0.36, -0.2)	<0.001	-1.61 (-1.81, -1.41)	<0.001	-2.28 (-2.51, -2.05)	<0.001
Region								
Northeast	Ref.	Ref.	Ref.	Ref.	Ref.	Ref.	Ref.	Ref.
Midwest	-0.08 (-0.19,0.03)	0.159	-0.6 (-0.63, -0.56)	<0.001	0.24 (0.12,0.36)	<0.001	0.13 (0.01,0.25)	0.038

© 2025 Callaway Kim K et al. *JAMA Health Forum*.

South	-0.88 (-0.99, -0.77)	<0.001	0.47 (0.43,0.51)	<0.001	0.82 (0.70,0.92)	<0.001	0.72 (0.59,0.84)	<0.001
West	-0.92 (-1.05, -0.80)	<0.001	-0.27 (-0.31, -0.23)	<0.001	0.48 (0.35,0.61)	<0.001	0.53 (0.39,0.66)	<0.001
Missing/unknown	-1.23 (-1.37, -1.09)	<0.001	-0.03 (-0.14, -0.02)	0.283	0.24 (0.10,0.38)	0.001	0.32 (0.17,0.46)	<0.001
Patient residential location based on 3-level ZIP code								
<50% metropolitan by population	Ref.	Ref.	Ref.	Ref.	Ref.	Ref.	Ref.	Ref.
≥50% metropolitan by population	-0.25 (-0.32, -0.18)	<0.001	0.18 (0.16,0.20)	<0.001	0.22 (0.14,0.3)	<0.001	0.23 (0.14,0.31)	<0.001
Missing/unknown	-0.52 (-0.61, -0.44)	<0.001	0.20 (0.18,0.23)	<0.001	0.38 (0.29,0.47)	<0.001	0.45 (0.36,0.54)	<0.001
Pre-recall PDC category								
Low (<50 p.p.)	Ref.	Ref.	Ref.	Ref.	Ref.	Ref.	Ref.	Ref.
Moderate (51-79 p.p.)	20.71 (20.40,21.02)	<0.001	0.37 (0.34,0.40)	<0.001	-12.93 (-13.24, -12.62)	<0.001	--15.04 (-15.42, --14.65)	<0.001
High (≥80 p.p.)	39.08 (38.78,39.38)	<0.001	0.91 (0.87,0.94)	<0.001	-26.66 (-26.95, -26.36)	<0.001	-27.27 (-27.64, -26.90)	<0.001
Most common fill location pre-recall								
Retail pharmacy	Ref.	Ref.	Ref.	Ref.	Ref.	Ref.	Ref.	Ref.
Mail-order pharmacy	-0.32 (-0.41, -0.23)	<0.001	-0.87 (-0.9, -0.84)	<0.001	1.80 (1.71,1.89)	<0.001	0.71 (0.62,0.81)	<0.001
Long term care facility	-0.55 (-0.63, -0.48)	<0.001	-0.43 (-0.46, -0.4)	<0.001	-1.66 (-1.74, -1.59)	<0.001	-1.18 (-1.26, -1.11)	<0.001
Unknown/mixed/missing	-0.86 (-0.94, -0.78)	<0.001	1.57 (1.53,1.61)	<0.001	1.1 (1.01,1.19)	<0.001	0.81 (0.72,0.90)	<0.001
Insurance type on last pre-recall fill								
Cash/self-pay	Ref.	Ref.	Ref.	Ref.	Ref.	Ref.	Ref.	Ref.
Medicaid fee-for-service	0.85 (0.72,0.98)	<0.001	0.54 (0.51,0.57)	<0.001	-0.72 (-0.87, -0.58)	<0.001	-0.53 (-0.71, -0.36)	<0.001
Third-party	3.30 (3.17,3.43)	<0.001	0.70 (0.68,0.73)	<0.001	-1.89 (-2.03, -1.74)	<0.001	-2.02 (-2.18, -1.85)	<0.001
Medicare	4.03 (3.9,4.17)	<0.001	0.95 (0.92,0.98)	<0.001	-2.04 (-2.19, -1.89)	<0.001	-2.46 (-2.63, -2.29)	<0.001

© 2025 Callaway Kim K et al. *JAMA Health Forum*.

Pre-recall anti-hypertension medication use category								
1 drug class	Ref.	Ref.	Ref.	Ref.	Ref.	Ref.	Ref.	Ref.
2 drug classes	0.14 (0.03,0.25)	0.015	0.54 (0.50,0.57)	<0.001	-0.18 (-0.30, -0.06)	0.004	-0.22 (-0.34, -0.09)	0.001
3 drug classes	-0.22 (-0.32, -0.11)	<0.001	1.53 (1.49,1.57)	<0.001	-0.07 (-0.19,0.05)	0.228	-0.10 (-0.22,0.02)	0.106
≥4 drug classes	-0.79 (-0.90, -0.67)	<0.001	3.15 (3.10,3.20)	<0.001	0.15 (0.03,0.27)	0.016	0.21 (0.09,0.34)	0.001
Primary prescriber specialty pre-recall								
Internal medicine or other general practice	Ref.	Ref.	Ref.	Ref.	Ref.	Ref.	Ref.	Ref.
Physician's assistant or nurse practitioner	-0.72 (-0.82, -0.62)	<0.001	0.01 (-0.02,0.04)	0.512	0.29 (0.18,0.40)	<0.001	0.45 (0.34,0.57)	<0.001
Cardiovascular disease	0.12 (0.03,0.20)	0.008	0.52 (0.48,0.56)	<0.001	-0.26 (-0.35, -0.17)	<0.001	-0.27 (-0.37, -0.18)	<0.001
Nephrology	-0.77 (-0.86, -0.69)	<0.001	0.67 (0.63,0.71)	<0.001	0.20 (0.11,0.29)	<0.001	0.28 (0.19,0.39)	<0.001
Diabetes, endocrine, or other metabolic disease	-0.13 (-0.23, -0.02)	0.016	0.32 (0.29,0.36)	<0.001	0.32 (0.21,0.44)	<0.001	0.27 (0.15,0.39)	<0.001
Other specialty	-1.03 (-1.12, -0.93)	<0.001	0.11 (0.08,0.14)	<0.001	0.26 (0.16,0.36)	<0.001	0.50 (0.39,0.60)	<0.001
Missing/unknown	-1.53 (-1.64, -1.43)	<0.001	0.05 (0.01,0.08)	0.009	0.40 (0.28,0.51)	<0.001	0.66 (0.53,0.79)	<0.001

Abbreviations: ARB = angiotensin-receptor-II blocker; PDC = proportion of days covered; p.p. = percentage points; Ref. = reference

© 2025 Callaway Kim K et al. *JAMA Health Forum*.

e-Table 4: Full Difference-in-Difference Models for Adjusted Changes in Drug Spending Outcomes Post- vs. Pre-Recall, Established ARB vs. Comparison Drug Users

e-Table 4 presents the full output from our main difference-in-difference models for drug spending outcomes.

Variable	Mean Insurer Spending per Patient-Quarter		Mean OOP Spending per Patient-Quarter		Generic Fills per Patient-Quarter		Rejected Fills per Patient-Quarter	
	Estimate (95% CI)	p-value	Estimate (95% CI)	p-value	Estimate (95% CI)	p-value	Estimate (95% CI)	p-value
Intercept	-79.76 (-81.05, -78.46)	<0.001	53.01 (52.25,53.78)	<0.001	1.15 (1.14,1.16)	<0.001	-0.01 (-0.01, -0.009)	<0.001
ARB vs. comparison drug user at baseline	56.55 (55.6,57.50)	<0.001	8.1 (7.92,8.29)	<0.001	-0.09 (-0.1, -0.09)	<0.001	0.001 (0.001,0.001)	<0.001
First Quarter of Valsartan Recall (7/13/18 – 10/10/18)	-1.92 (-2.61, -1.23)	<0.001	-1.81 (-1.98, -1.64)	<0.001	-0.1 (-0.10, -0.09)	<0.001	-0.004 (-0.004, -0.003)	<0.001
ARB user*Post-Valsartan-Recall	0.79 (-1.05,2.64)	0.400	0.95 (0.56,1.33)	<0.001	0.05 (0.04,0.06)	<0.001	0.01 (0.01,0.01)	<0.001
After First Losartan and Irbesartan Recalls (10/11/18 – 1/3/20)	3.17 (2.72,3.61)	<0.001	-1.59 (-1.71, -1.48)	<0.001	-0.15 (-0.15, -0.14)	<0.001	0.002 (0.001,0.002)	<0.001
ARB user*Post-Losartan-Irbesartan-Recalls	2.23 (1.02,3.44)	<0.001	-0.53 (-0.76, -0.3)	<0.001	0.05 (0.04,0.05)	<0.001	0.002 (0.002,0.002)	<0.001
Female gender	-0.67 (-1.12, -0.22)	0.004	-1.31 (-1.4, -1.22)	<0.001	-0.01 (-0.01, -0.01)	<0.001	0.004 (0.004,0.005)	<0.001
Age group in 2018								
<20	Ref.	Ref.	Ref.	Ref.	Ref.	Ref.	Ref.	Ref.
20-29	2.70 (1.77,3.62)	<0.001	-1.08 (-1.37, -0.78)	<0.001	-0.12 (-0.13, -0.11)	<0.001	0.001 (0,0.002)	0.067
30-39	10.19 (9.29,11.09)	<0.001	0.27 (-0.03,0.57)	0.075	-0.2 (-0.21, -0.19)	<0.001	0.001 (0,0.002)	0.075
40-49	13.37 (12.49,14.24)	<0.001	1.73 (1.43,2.02)	<0.001	-0.24 (-0.25, -0.23)	<0.001	0.002 (0.001,0.003)	0.002
50-63	12.63 (11.77,13.50)	<0.001	2.2 (1.91,2.48)	<0.001	-0.27 (-0.28, -0.26)	<0.001	0.003 (0.002,0.004)	<0.001
65-79	10.33 (9.45,11.21)	<0.001	4.82 (4.54, 5.11)	<0.001	-0.41 (-0.40, -0.40)	<0.001	-0.01 (-0.01, -0.01)	<0.001
≥80	4.99 (3.97,6.01)	<0.001	5.66 (5.36, 5.96)	<0.001	-0.37 (-0.38, -0.36)	<0.001	-0.01 (-0.01, -0.01)	<0.001
Region								
Northeast	Ref.	Ref.	Ref.	Ref.	Ref.	Ref.	Ref.	Ref.
Midwest	20.38 (19.65,21.11)	<0.001	-0.27 (-0.38, -0.15)	<0.001	-0.05 (-0.05, -0.05)	<0.001	0.004 (0.004,0.005)	<0.001

© 2025 Callaway Kim K et al. *JAMA Health Forum*.

South	20.19 (19.56,20.81)	<0.001	2.21 (2.09,2.33)	<0.001	-0.12 (-0.12, -0.11)	<0.001	0.01 (0.01,0.01)	<0.001
West	10.18 (9.55,10.80)	<0.001	-2.24 (-2.37, -2.11)	<0.001	0.06 (0.05,0.06)	<0.001	0.01 (0,0.01)	<0.001
Missing/unknown	-22.43 (-23.77, -21.10)	<0.001	-2.53 (-2.72, -2.33)	<0.001	-0.03 (-0.03, -0.02)	<0.001	-0.01 (-0.01, -0.01)	<0.001
Patient residential location based on 3-level ZIP code								
≥50% metropolitan by population	Ref.	Ref.	Ref.	Ref.	Ref.	Ref.	Ref.	Ref.
< 50% metropolitan by population	29.44 (29.02,29.87)	<0.001	-0.70 (-0.79, -0.61)	<0.001	-0.05 (-0.05, -0.05)	<0.001	0.005 (0.005,0.005)	<0.001
Missing/unknown	25.97 (25.42,26.51)	<0.001	-0.63 (-0.73, -0.52)	<0.001	-0.02 (-0.02, -0.01)	<0.001	0.01 (0.01,0.01)	<0.001
Pre-recall PDC category								
Low (<50 p.p.)	Ref.	Ref.	Ref.	Ref.	Ref.	Ref.	Ref.	Ref.
Moderate (51-79 p.p.)	18.65 (18.12,19.18)	<0.001	3.99 (3.87,4.12)	<0.001	0.18 (0.18,0.19)	<0.001	0.003 (0.003,0.003)	<0.001
High (≥80 p.p.)	32.61 (32.10,33.12)	<0.001	7.39 (7.27,7.52)	<0.001	0.34 (0.33,0.34)	<0.001	0.01 (0.01,0.01)	<0.001
Most common fill location pre-recall								
Retail pharmacy	Ref.	Ref.	Ref.	Ref.	Ref.	Ref.	Ref.	Ref.
Mail-order pharmacy	-66.35 (-67.06, -65.64)	<0.001	-15.77 (-15.89, -15.65)	<0.001	-0.39 (-0.40, -0.39)	<0.001	-0.04 (-0.04, -0.04)	<0.001
Long term care facility	-39.99 (-40.6, -39.38)	<0.001	0.63 (0.51,0.75)	<0.001	0.71 (0.70,0.73)	<0.001	-0.004 (-0.004, -0.003)	<0.001
Unknown/mixed/missing	-33.16 (-33.63, -32.68)	<0.001	-2.24 (-2.38, -2.09)	<0.001	-0.11 (-0.11, -0.11)	<0.001	0.01 (0.01,0.01)	<0.001
Insurance type on last pre-recall fill								
Cash/self-pay	Ref.	Ref.	Ref.	Ref.	Ref.	Ref.	Ref.	Ref.
Medicaid fee-for-service	87.73 (86.89,88.58)	<0.001	-54.02 (-54.78, -53.26)	<0.001	0.38 (0.37,0.38)	<0.001	0.09 (0.09,0.09)	<0.001
Third-party	58.19 (57.73,58.66)	<0.001	-40.06 (-40.84, -39.29)	<0.001	0.15 (0.14,0.15)	<0.001	0.04 (0.04,0.04)	<0.001
Medicare	72.83 (72.25,73.40)	<0.001	-49.41 (-50.17, -48.65)	<0.001	0.05 (0.05,0.05)	<0.001	0.06 (0.06,0.06)	<0.001

© 2025 Callaway Kim K et al. *JAMA Health Forum*.

Pre-recall anti-hypertension medication use category								
1 drug class	Ref.	Ref.	Ref.	Ref.	Ref.	Ref.	Ref.	Ref.
2 drug classes	3.901(3.39,4.42)	<0.001	0.62 (0.51,0.74)	<0.001	0.12 (0.11,0.12)	<0.001	0.01 (0.01,0.01)	<0.001
3 drug classes	22.24 (21.56,22.93)	<0.001	1.33 (1.21,1.45)	<0.001	0.25 (0.24,0.25)	<0.001	0.01 (0.01,0.01)	<0.001
≥4 drug classes	25.83 (25.12,26.55)	<0.001	0.78 (0.64,0.92)	<0.001	0.39 (0.39,0.4)	<0.001	0.02 (0.02,0.02)	<0.001
Primary prescriber specialty pre-recall								
Internal medicine or other general practice	Ref.	Ref.	Ref.	Ref.	Ref.	Ref.	Ref.	Ref.
Physician's assistant or nurse practitioner	-5.46 (-5.83, -5.1)	<0.001	-0.45 (-0.55, -0.35)	<0.001	0.06 (0.05,0.06)	<0.001	0.01 (0.01,0.01)	<0.001
Cardiovascular disease	40.33 (39.05,41.61)	<0.001	4.06 (3.93,4.19)	<0.001	0.06 (0.06,0.07)	<0.001	-0.005 (-0.005, -0.005)	<0.001
Nephrology	4.2 (3.69,4.71)	<0.001	-0.31 (-0.42, -0.2)	<0.001	0.18 (0.18,0.18)	<0.001	-0.003 (-0.004, -0.003)	<0.001
Diabetes, endocrine, or other metabolic disease	4.41 (3.87,4.95)	<0.001	-0.97 (-1.09, -0.85)	<0.001	0.1 (0.1,0.11)	<0.001	0.001 (0.001,0.001)	<0.001
Other specialty	6.42 (5.97,6.87)	<0.001	0.41 (0.31,0.51)	<0.001	0.07 (0.07,0.08)	<0.001	0.003 (0.003,0.003)	<0.001
Missing/unknown	-20.68 (-21.27, -20.08)	<0.001	-2.7 (-2.85, -2.56)	<0.001	0.09 (0.08,0.09)	<0.001	0.06 (0.05,0.06)	<0.001

Abbreviations: ARB = angiotensin-receptor-II blocker; PDC = proportion of drugs covered; p.p. = percentage points; Rx. = prescription; OOP = out-of-pocket; Ref. = reference

© 2025 Callaway Kim K et al. *JAMA Health Forum*.

e-Table 5: Reasons for Rejected Fills, by Period and Study Group

e-Table 5 describes listed reasons for rejected fills pre-recall, during the first 90 days after valsartan's first recall, and in the 5 quarters after irbesartan and losartan's first recalls.

Reason for Rejected Fill	Users of Recalled ARBS at Baseline, No. Rejected Fills (% of Rejected)			Users of Comparison Drugs at Baseline, No. Rejected Fills (% of Rejected)		
	Pre-Recall (7/18/17 – 7/12/18)	First Quarter of Valsartan Recall (7/13/18 – 10/10/18)	After First Irbesartan and Losartan Recalls (10/11/18 – 1/3/20)	Pre-Recall (7/18/17 – 7/12/18)	First Quarter of Valsartan Recall (7/13/18 – 10/10/18)	After First Irbesartan and Losartan Recalls (10/11/18 – 1/3/20)
Refill restriction	1,673,531 (52.8%)	473,514 (54.6%)	2,569,677 (67.2%)	2,728,639 (53.3%)	764,821 (62.0%)	4,144,842 (69.4%)
Step edit	9689,669 (31.2%)	199,115 (23.0%)	560,482 (14.7%)	1,565,746 (30.6%)	292,115 (23.7%)	888,033 (14.9%)
Missing or invalid information on claim	249,327 (7.9%)	97,004 (11.2%)	385,668 (10.1%)	445,304 (8.7%)	101,717 (8.3%)	575,491 (9.6%)
Product not covered	106,231 (3.4%)	53,584 (6.2%)	143,652 (3.8%)	170,500 (3.3%)	32,445 (2.6%)	163,107 (2.7%)
Plan limitations exceeded	92,933 (2.9%)	29,129 (3.4%)	115,798 (3.0%)	144,596 (2.8%)	30,350 (2.5%)	42,083 (2.4%)
Prior authorization required	40,174 (1.3%)	8,966 (1.0%)	32,855 (0.9%)	27,318 (0.5%)	5,829 (0.5%)	31,601 (0.5%)
Distribution limitation	15,182 (0.5%)	3,245 (0.4%)	14,878 (0.4%)	29,590 (0.6%)	5,303 (0.4%)	24,371 (0.4%)
Missing reject code	2,831 (0.1%)	15 (<0.01%)	1 (<0.01%)	4,326 (0.1%)	19 (<0.01%)	1 (<0.01%)

Abbreviations: ARB = angiotensin-receptor-II blocker; No. = number

© 2025 Callaway Kim K et al. *JAMA Health Forum*.

e-Table 6: Baseline Characteristics of Established ARB and Comparison Drug Users, Sensitivity Analysis Using Individual-level Data for 20% Random Sample

e-Table 6 presents descriptive statistics on characteristics of a 20% random sample for a sensitivity analysis using individual-level data.

Variable	Full Sample			20% Random Sample		
	Users of Recalled ARBs at Baseline	Users of Comparison Drugs at Baseline	Std. Diff.	Users of Recalled ARBs at Baseline	Users of Comparison Drugs at Baseline	Std. Diff.
	13,755,251 (100)	23,426,296 (100)		3,438,489 (100)	5,856,774 (100)	
Most-recent pre-recall fill						
Valsartan	3,147,530 (22.9)	NA		787,106 (22.9)	NA	
Irbesartan	671,438 (4.9)	NA		167,328 (4.9)	NA	
Losartan	9,936,283 (72.2)	NA		2,484,055 (72.2)	NA	
ACE-I	NA	22,147,622 (94.5)		NA	5,2537,718 (94.6)	
Non-recalled ARB[a]	NA	1,278,674 (5.5)		NA	319,056 (5.4)	
Median (IQR) age in 2018	66 (56,74)	62 (54,72)	0.1808	66 (56,74)	63 (54,72)	0.1817
Age group in 2018			0.1814			0.1814
18-19	7,069 (0.1)	26,710 (0.1)		1,807 (0.1)	6,862 (0.1)	
20-29	77,126 (0.6)	217,469 (0.9)		19,220 (0.6)	54,439 (0.9)	
30-39	373,762 (2.8)	930,796 (4.1)		93,144 (2.7)	232,893 (4.0)	
40-49	1,248,347 (9.3)	2,653,751 (11.7)		312,431 (9.1)	663,787 (11.3)	
50-64	4,729,740 (34.4)	8,975,948 (38.3)		1,181,279 (34.3)	2,242,973 (38.3)	
65-79	5,384,236 (39.1)	8,092,124 (34.5)		1,346,550 (39.2)	2,023,832 (34.6)	
≥80	1,934,971 (14.1)	2,529,498 (10.8)		484,058 (14.1)	631,988 (10.8)	
Gender						
Female	7,533,747 (54.8)	10,773,448 (46.0)	0.1763	1,883,149 (54.8)	2,693,230 (46.0)	0.1763
Male	6,221,504 (45.2)	12,652,848 (54.0)		1,555,340 (45.2)	3,163,544 (54.0)	
Region			0.1016			0.1016
Northeast	2,400,137 (17.4)	3,876,451 (16.5)		599,445 (17.4)	969,401 (16.6)	
Midwest	2,719,913 (19.8)	5,348,442 (22.8)		679,722 (19.8)	1,337,066 (22.8)	
South	5,680,747 (41.3)	9,487,052 (40.5)		1,420,387 (41.3)	2,371,566 (40.5)	
West	2,520,184 (18.3)	4,032,751 (17.2)		630,069 (18.3)	1,007,997 (17.2)	

© 2025 Callaway Kim K et al. *JAMA Health Forum*.

Missing/unknown	34,270 (3.2)	681,600 (2.9)		108,866 (3.2)	170,744 (2.9)	
Patient residential location based on 3-level ZIP code			0.0694			0.0694
<50% metropolitan by population	1,389,755 (10.1)	2,858,347 (12.2)		348,005 (10.1)	714,331 (12.2)	
≥50% metropolitan by population[b]	11,017,935 (80.1)	8,448,542 (78.8)		2,753,678 (80.1)	4,612,265 (78.8)	
Missing/unknown	1,47,561 (9.8)	2,119,407 (9.0)		336,806 (9.8)	530,178 (9.1)	
Median (IQR) pre-recall PDC	91.1 (76.6,97.1)	90.2 (75.0,96.8)	0.0650	91.1 (76.6,97.1)	90.2 (75.0,96.8)	0.0650
Pre-recall PDC category			0.0528			0.0528
Low (<50 p.p.)	21,186 (4.5)	1,300,660 (5.6)		154,915 (4.5)	325,591 (5.6)	
Moderate (51-79 p.p.)	3,436,714 (25.0)	6,157,964 (26.3)		860,019 (25.0)	1,539,535 (26.3)	
High (≥80 p.p.)	9,697,351 (70.5)	15,967,672 (68.2)		2,423,555 (70.5)	3,991,648 (68.2)	
Most common fill location pre-recall			0.0836			0.0836
Retail pharmacy	11,627,233 (84.5)	20,119,469 (85.9)		2,906,673 (84.5)	5,030,421 (85.9)	
Mail-order pharmacy	1,466,405 (10.7)	2,203,769 (9.4)		366,254 (10.7)	550,445 (9.4)	
Long term care facility	108,819 (0.8)	203,726 (0.9)		27,354 (0.8)	51,010 (0.9)	
Unknown/mixed/missing	52,794 (4.0)	99,332 (3.8)		138,208 (4.0)	224,898 (3.8)	
Insurance type on last pre-recall fill			0.2386			0.2386
Medicare[c]	6,122,101 (44.5)	8,940.581 (38.2)		1,530,675 (44.5)	2,234,021 (38.1)	
Third-Party[d]	7,306,139 (53.1)	13,221,684 (56.4)		1825,916 (53.1)	3,305,972 (56.4)	
Medicaid fee-for-service	19,892 (1.0)	383,420 (1.6)		35,064 (1.0)	95,992 (1.6)	
Cash/self-pay	187,119 (1.4)	880,611 (3.8)		46,834 (1.4)	220,789 (3.8)	
Primary prescriber specialty pre-recall[e]			0.2136			0.1755
Internal medicine or other general practice	9,105,323 (66.2)	15,211,694 (64.9)		2,275,562 (66.2)	3,802,333 (64.9)	
Physician's assistant or nurse practitioner	2,127,742 (15.5)	4,587,815 (19.6)		531,633 (15.5)	1,148,011 (19.6)	
Cardiovascular disease	1528,836 (11.1)	1,994,061 (8.5)		383,228 (11.1)	497,724 (8.5)	
Nephrology	29,122 (1.8)	283,479 (1.2)		62,444 (1.8)	71,023 (1.2)	
Diabetes, endocrine, or other metabolic disease	51,667 (1.1)	248,479 (1.1)		37,623 (1.1)	62,129 (1.1)	
Other specialty	499,340 (3.6)	894,935 (3.8)		124,725 (3.6)	223,943 (3.8)	
Missing/unknown	93,221 (0.7)	205,872 (0.9)		23,274 (0.7)	51,611 (0.9)	

© 2025 Callaway Kim K et al. *JAMA Health Forum*.

Pre-recall anti-hypertension medication use category			0.1509			0.1509
1 drug class	4,835,190 (35.2)	10,254,229 (43.8)		1,207,504 (35.1)	2,564,762 (43.8)	
2 drug classes	4,713,553 (34.3)	7,664,579 (32.7)		1,178,720 (34.3)	1,915,662 (32.7)	
3 drug classes	3,013,280 (21.9)	4,106,274 (17.5)		754,639 (21.9)	1,026,251 (17.5)	
≥4 drug classes	1,193,228 (8.7)	1401,223 (6.0)		297,626 (8.7)	350,099 (6.0)	
Pre-recall use of other anti-hypertension classes[f]						
Calcium channel blockers	4,554,287 (33.1)	5,624,094 (24.0)	0.2025	1,139,212 (33.1)	1,406,401 (24.0)	0.2029
Diuretics	4,053,268 (29.5)	6,110,257 (26.1)	0.0756	1,012,954 (29.5)	1,526,434 (26.1)	0.0759
Adrenergic blockers	5,416,054 (39.4)	8,045,544 (34.3)	0.1044	1,354,880 (39.4)	2,010,655 (34.3)	0.1053
Other renin-angiotensin agent	32,929 (0.2)	29,155 (0.1)	0.0270	8,179 (0.2)	7,281 (0.1)	0.0267
Other drug class	406,030 (3.0)	412,819 (1.8)	0.0785	101,217 (2.9)	103,000 (1.8)	0.0783

Abbreviations: ARB = angiotensin-II-receptor blocker; Std. Diff. = standardized difference; PDC = proportion of days covered; p.p. = percentage points; IQR = interquartile range

a. Non-recalled ARBs included azilsartan, candesartan, eprosartan, olmesartan, and telmisartan, including combination products (see e-Table 1 in Supplement).
b. Areas were defined as predominantly metropolitan if ≥50% of the population resided in ZIP codes classified as 2010 Rural Urban Commuting Areas “1” (Metropolitan area core), “2” (Metropolitan area high commuting) or “3” (Metropolitan area low commuting).
c. Medicare insurance type included both Medicare and Medicare Part D.
d. Third party insurance included coverage from an employer, Medicaid Managed Care, and individual market plans. We did not have access to reliable variables to distinguish between these types.
e. Patients were assigned a single primary prescriber based on the most fills in the pre-recall period (7/18/2017 – 7/12/2018). See e-Table 1 in Supplement for codes used to define each prescriber specialty.
f. Specific drugs included in each anti-hypertension class can be found in e-Table 1 in the Supplement.

© 2025 Callaway Kim K et al. *JAMA Health Forum*.

e-Table 7: Adjusted Differential Changes in Outcomes for Established ARB vs. Comparison Drug Users, Post- vs. Pre-Recall, Sensitivity Analysis Using Individual-level Data for 20% Random Sample

e-Table 7 presents the main difference-in-different effect estimates from a sensitivity analysis using individual-level data on a 20% random sample.

Outcome	Pre-Recall Use (7/18/2017 – 7/12/2018)			Difference-in-Difference Estimates[a]					
				First Quarter of Valsartan Recall (7/13/2018 – 10/10/2018)			After First Losartan and Irbesartan Recalls (10/11/2018 – 1/3/2020)		
	Users of Recalled ARBs at Baseline	Users of Comparison Drugs at Baseline	Diff.	Coef. (95% CI)	p-value	Rel. change[b]	Coef. (95% CI)	p-value	Rel. change[b]
Mean PDC (p.p.) per patient-quarter	85.3	84.4	0.9	0.32 (0.29, 0.36)	<0.001	0.4%	-0.13 (-0.16, -0.10)	<0.001	-0.2%
% with Rx. Switch	3.8	1.5	2.3	8.24 (8.21, 8.26)	<0.001	217%	1.00 (0.98, 1.02)	<0.001	26.3%
% with Rx. Gap ≥30 days	10.0	10.8	-0.8	-0.30 (-0.34, -0.26)	<0.001	-3.0%	0.42 (0.39, 0.44)	<0.001	4.2%
Mean days between fills	24.9	26.2	-1.3	-0.51 (-0.55, -0.46)	<0.001	-2.0%	0.50 (0.48, 0.53)	<0.001	2.0%
Mean Payer Rx. Spending per patient-quarter	123.2	62.7	60.4	-0.95 (-1.2, -0.74)	<0.001	-0.8%	-3.4 (-3.6, -3.2)	<0.001	-2.8%
Mean OOP Rx. Spending per patient-quarter	25.5	18.7	6	*Did not converge*					
Generic fills	1.2	1.2	0.0	0.058 (0.057, 0.059)	<0.001	4.8%	0.044 (0.043, 0.045)	<0.001	3.7%
Rejected fills	0.061	0.058	0.003	0.007 (0.007, 0.008)	<0.001	11.4%	0.002 (0.0016, 0.002)	<0.001	3.3%

Abbreviations: ARB = angiotensin-receptor-II blocker; PDC = proportion of days covered; p.p. = percentage points; Rx. = prescription; OOP = out-of-pocket; Diff. = difference; Coef. = coefficient; CI = confidence interval; Rel. = relative

a. Difference-in-difference estimates for the intention-to-treat differential change in outcomes for ARB versus comparison drug users. Models were adjusted for age group (19-20, 20-29, 30-39, 40-49, 50-64, 65-79, ≥80 years), female gender, pre-recall fill location (retail, long-term-care, mail-order, or unknown/mixed), pre-recall anti-hypertension medication use category (1, 2, 3, or ≥4 drug classes), pre-recall PDC category (<50, 51-79, ≥80 p.p.), primary prescriber specialty, U.S. geographic region, patient metropolitan residential location and insurance type (Medicare, Third party, Medicaid fee-for-service, or Cash/self-pay). For the individual-level analysis, we used generalized estimating equations (GEEs) with a gaussian distribution and identity link to estimate linear changes while accounting for correlated intra-person observations. We assumed unstructured correlation within patient-quarters.

b. Relative changes were calculated as the difference-in-difference estimate, divided by the mean quarterly use among baseline ARB users in the pre-recall period (7/18/2017 – 7/12/2018).

© 2025 Callaway Kim K et al. *JAMA Health Forum*.

e-Table 8: Differential Changes in Medication Use Outcomes, Sensitivity Analyses Using Different Cohort Inclusion Criteria

e-Table 8 presents results from a difference-in-difference sensitivity analysis for differential changes in medication use outcomes from sensitivity analyses using different cohort inclusion criteria.

Outcome	No. Patients		Mean Pre-Recall Use per Quarter (7/18/2017 – 7/12/2018)			Difference-in-Difference Estimates[a]					
						First Quarter of Valsartan Recall (7/13/18 – 10/10/18)			After First Irbesartan and Losartan Recalls (10/11/18 – 1/3/20)		
	Users of Recalled ARBs at Baseline	Users of Comparison Drugs at Baseline	ARB Users	Comparison Drug Users	Diff.	Coef. (95% CI)	p-value	Rel. change[b]	Coef. (95% CI)	p-value	Rel. change[b]
Mean PDC (p.p.) per patient-quarter											
Main cohort	13,755,251	23,426,296	85.62	84.60	1.02	0.55 (0.34,0.76)	<0.001	0.6%	-0.17 (-0.37,0.02)	0.087	-0.2%
Relax rx. requirement[c]	14,906,198	25,721.357	83.79	82.46	1.34	0.61 (0.43,0.8)	<0.001	0.7%	-0.24 (-0.44, -0.04)	0.019	-0.3%
Exclude LTC users	13,646,432	23,222,570	85.63	84.61	1.02	0.56 (0.35,0.77)	<0.001	0.7%	-0.17 (-0.37,0.03)	0.087	-0.2%
Exclude ACE-I users	13,755,251	1,278,674	85.62	85.31	0.31	0.6 (0.38,0.82)	<0.001	0.7%	0.59 (0.37,0.80)	<0.001	0.7%
% With Rx. Switch per patient-quarter											
Main cohort	13,755,251	23,426,296	3.70	1.59	2.1	8.46 (8.3,8.62)	<0.001	229.0%	1.2 (1.12,1.27)	<0.001	32.4%
Relax rx. requirement[c]	14,906,198	25,721,357	3.65	1.57	2.08	8.19 (8.02,8.36)	<0.001	2274.4%	1.17 (1.1,1.24)	<0.001	32.1%
Exclude LTC users	13,646,432	23,222,570	3.70	1.59	2.1	8.46 (8.30,8.63)	<0.001	228.8%	1.2 (1.13,1.28)	<0.001	32.6%
Exclude ACE-I users	13,755,251	1,278,674	3.70	4.69	-0.99	9.9 (9.7,10.09)	<0.001	267.7%	-0.18 (-0.33, -0.02)	0.028	-4.9%
% With Rx. Gap ≥30 days per patient-quarter											
Main cohort	13,755,251	23,426,296	9.45	10.24	-0.79	-0.24 (-0.46, -0.03)	0.03	-2.5%	0.35 (0.14,0.56)	0.001	3.7%
Relax rx. requirement[c]	14,906,198	25,721,357	10.01	10.87	-0.86	-0.16 (-0.37,0.06)	0.16	-1.5%	0.35 (0.15,0.55)	0.001	3.5%

© 2025 Callaway Kim K et al. *JAMA Health Forum*.

Exclude LTC users	13,646,432	23,222,570	9.46	10.25	-0.80	-0.24 (-0.45, -0.03)	0.028	-2.5%	0.35 (0.15,0.56)	0.001	3.7%
Exclude ACE-I users	13,755,251	1,278,674	9.45	9.90	-0.45	-0.34 (-0.57, -0.11)	0.003	-3.6%	-0.3 (-0.53, -0.07)	0.011	-3.2%
Mean days between fills per patient-quarter											
Main cohort	13,755,251	23,426,296	10.61	10.61	-0.94	-0.28 (-0.46, -0.1)	0.003	-2.6%	0.51 (0.29,0.72)	<0.001	4.8%
Relax rx. requirement[c]	14,906,198	25,721,357	12.79	12.79	-1.25	0.05 (-0.18,0.28)	0.67	0.4%	0.73 (0.49,0.97)	<0.001	5.7%
Exclude LTC users	13,646,432	23,222,570	10.61	10.61	-0.95	-0.28 (-0.47, -0.09)	0.003	-2.6%	0.51 (0.29,0.72)	<0.001	4.8%
Exclude ACE-I users	13,755,251	1,278,674	10.61	10.61	-0.63	-0.39 (-0.60, -0.187	<0.001	-3.7%	-0.15 (-0.38,0.08)	0.201	-1.4%

Abbreviations: ARB = angiotensin-receptor-II blocker; PDC = proportion of days covered; p.p. = percentage points; Rx. = prescription; LTC = long-term care; ACE-I=angiotensin-converting-enzyme inhibitor; Diff. = difference; Coef. = coefficient; CI = confidence interval; Rel. = relative

a. Difference-in-difference estimates for the intention-to-treat differential change in outcomes for ARB versus comparison drug users. Models were adjusted for age group (19-20, 20-29, 30-39, 40-49, 50-64, 65-79, ≥80 years), female gender, pre-recall fill location (retail, long-term-care, mail-order, or unknown/mixed), pre-recall anti-hypertension medication use category (1, 2, 3, or ≥4 drug classes), pre-recall PDC category (<50, 51-79, ≥80 p.p.), primary prescriber specialty, U.S. geographic region, patient metropolitan residential location and insurance type (Medicare, Third party, Medicaid fee-for-service, or Cash/self-pay).
b. Relative changes were calculated as the difference-in-difference estimate, divided by the mean quarterly use among baseline ARB users in the pre-recall period (7/18/2017 – 7/12/2018).
c. Sensitivity analysis for which we did not require medication possession in the quarter immediately prior to valsartan's recall.

© 2025 Callaway Kim K et al. *JAMA Health Forum*.

e-Table 9: Differential Changes in Drug Spending Outcomes, Sensitivity Analyses Using Different Cohort Inclusion Criteria

e-Table 9 presents results from a difference-in-difference sensitivity analysis for differential changes in drug spending outcomes from sensitivity analyses using different cohort inclusion criteria.

Outcome	No. Patients		Mean Pre-Recall Use per Quarter (7/18/2017 – 7/12/2018)			Difference-in-Difference Estimates[a]					
						First Quarter of Valsartan Recall (7/13/18 – 10/10/18)			After First Irbesartan and Losartan Recalls (10/11/18 – 1/3/20)		
	Users of Recalled ARBs at Baseline	Users of Comparison Drugs at Baseline	ARB Users	Compariso n Drug Users	Diff.	Coef. (95% CI)	p-value	Rel. change[b]	Coef. (95% CI)	p-value	Rel. change[b]
Mean Payer Rx. Spending per patient-quarter											
Main cohort	13,755,251	23,426,296	122.60	61.50	61.04	0.79 (-1.05,2.64)	0.4	0.6%	2.23 (1.02,3.44)	<0.001	1.8%
Relax rx. requirement[c]	14,906,198	25,721,357	119.45	59.58	59.87	1.02 (-0.81,2.85)	0.274	0.9%	2.81 (1.62, 4.01)	<0.001	2.4%
Exclude LTC users	13,646,432	24,222,570	123.09	61.74	61.32	0.76 (-1.1,2.61)	0.423	0.6%	2.15 (0.94,3.36)	0.001	1.7%
Exclude ACE-I users	13,755,251	1,278,674	122.60	360.27	-238	28.45 (21.62,35.28)	<0.001	23.2%	51.24 (46.91,55.57)	<0.001	41.8%
Mean OOP Rx. Spending per patient-quarter											
Main cohort	13,755,251	23,426,296	25.32	18.41	6.91	0.95 (0.56,1.33)	<0.001	3.7%	-0.53 (-0.76, -0.30)	<0.001	-2.1%
Relax rx. requirement[c]	14,906,198	25,721,357	25.92	17.98	6.94	0.85 (0.47,1.22)	<0.001	3.4%	-0.59 (-0.81, -0.36)	<0.001	-2.1%
Exclude LTC users	13,646,432	23,222,570	25.31	18.41	6.90	0.95 (0.56,1.34)	<0.001	3.8%	-0.53 (-0.76, -0.30)	<0.001	-2.0%
Exclude ACE-I users	13,755,251	1,278,674	25.32	75.62	-50.3	12.18 (11.14,13.21)	<0.001	48.1%	14.75 (14.15,15.51)	<0.001	58.3%
Generic fills per patient-quarter											
Main cohort	13,755,251	23,426,296	1.13	1.21	-0.09	0.05 (0.04,0.06)	<0.001	4.7%	0.05 (0.04,0.05)	<0.001	4.3%
Relax rx. requirement[c]	14,906,198	25,721,357	1.11	1.19	-0.08	0.05 (0.04,0.06)	<0.001	4.8%	0.05 (0.04,0.05)	<0.001	4.1%
Exclude LTC users	13,646,432	23,222,570	1.12	1.21	-0.09	0.05 (0.04,0.06)	<0.001	4.7%	0.05 (0.04,0.05)	<0.001	4.3%
Exclude ACE-I users	13,755,251	1,278,674	1.13	0.80	0.33	0.0001 (-0.0117,0.0115)	0.987	0.0%	-0.08 (-0.09, -0.07)	<0.001	-7.0%

© 2025 Callaway Kim K et al. *JAMA Health Forum*.

Rejected fills per patient-quarter											
Main cohort	13,755,251	23,426,296	0.06	0.06	0	0.01 (0.01,0.01)	<0.001	11.9%	0.002 (0.001,0.002)	<0.001	3.2%
Relax rx. requirement[c]	14,906,198	25,721,357	0.06	0.06	0	0.007 (0.007,0.008)	<0.001	11.7%	0.002 (0.001,0.002)	<0.001	3.1%
Exclude LTC users	13,646,432	23,222,570	0.06	0.06	0	0.007 (0.007,0.008)	<0.001	11.9%	0.002 (0.002,0.002)	<0.001	3.2%
Exclude ACE-I users	13,755,251	1,278,674	0.06	0.07	-0.01	0.015 (0.014,0.015)	<0.001	23.6%	0.006 (0.005,0.006)	<0.001	9.2%

Abbreviations: ARB = angiotensin-receptor-II blocker; Rx. = prescription; OOP = out-of-pocket; LTC = long-term care; ACE-I=angiotensin-converting-enzyme inhibitor; Diff. = difference; Coef. = coefficient; CI = confidence interval; Rel. = relative

a. Difference-in-difference estimates for the intention-to-treat differential change in outcomes for ARB versus comparison drug users. Models were adjusted for age group (19-20, 20-29, 30-39, 40-49, 50-64, 65-79, ≥80 years), female gender, pre-recall fill location (retail, long-term-care, mail-order, or unknown/mixed), pre-recall anti-hypertension medication use category (1, 2, 3, or ≥4 drug classes), pre-recall PDC category (<50, 51-79, ≥80 p.p.), primary prescriber specialty, U.S. geographic region, patient metropolitan residential location and insurance type (Medicare, Third party, Medicaid fee-for-service, or Cash/self-pay).
b. Relative changes were calculated as the difference-in-difference estimate, divided by the mean quarterly use among ARB users in the pre-recall period (7/18/2017 – 7/12/2018).
c. Sensitivity analysis for which we did not require medication possession in the quarter immediately prior to valsartan's recall.

© 2025 Callaway Kim K et al. *JAMA Health Forum*.

e-Table 10: Sensitivity Analyses Using Varying Prescription Lengths to Define Medication Gaps

e-Table 10 presents the main difference-in-difference effect estimates from sensitivity analyses using 15, 45, 60 and 90 (versus 30) days to define medication gaps.

Outcome	Pre-Recall Use, (7/18/2017 – 7/12/2018)			Difference-in-Difference Estimates[a]					
				First Quarter of Valsartan Recall (7/13/18 – 10/10/18)			After First Irbesartan and Losartan Recalls (10/11/18 – 1/3/20)		
	Users of Recalled ARBs at Baseline	Users of Comparison Drugs at Baseline	Diff.	Coef. (95% CI)	p-value	Rel. change[b]	Coef. (95% CI)	p-value	Rel. change[b]
Gaps ≥30 days (primary)	9.45	10.24	-0.79	-0.24 (-0.45, -0.03)	0.028	-2.5%	0.35 (0.14,0.57)	0.001	3.7%
Gaps ≥15 days	16.63	17.79	-1.17	-0.3 (-0.54, -0.04)	0.024	-1.7%	0.50 (0.25,0.74)	<0.001	3.0%
Gaps ≥45 days	6.40	6.99	-0.59	-0.18 (-0.37,0)	0.052	-2.9%	0.26 (0.09,0.43)	0.003	4.0%
Gaps ≥60 days	4.51	5.01	-0.5	-0.13 (-0.29,0.03)	0.116	-2.8%	0.18 (0.03,0.33)	0.022	4.0%
Gaps ≥90 days	2.44	2.79	-0.35	-0.07 (-0.15,0.02)	0.112	-2.8%	0.14 (0.06,0.22)	0.001	5.6%

Abbreviations: ARB = angiotensin-receptor-II blocker; Diff. = difference; Coef. = coefficient; CI = confidence interval; Rel. = relative

a. Difference-in-difference estimates for the intention-to-treat differential change in outcomes for ARB versus comparison drug users. Models were adjusted for age group (19-20, 20-29, 30-39, 40-49, 50-64, 65-79, ≥80 years), female gender, pre-recall fill location (retail, long-term-care, mail-order, or unknown/mixed), pre-recall anti-hypertension medication use category (1, 2, 3, or ≥4 drug classes), pre-recall PDC category (<50, 51-79, ≥80 p.p.), primary prescriber specialty, U.S. geographic region, patient metropolitan residential location and insurance type (Medicare, Third party, Medicaid fee-for-service, or Cash/self-pay).
b. Relative changes were calculated as the difference-in-difference estimate, divided by the mean quarterly use among ARB users in the pre-recall period (7/18/2017 – 7/12/2018).

© 2025 Callaway Kim K et al. *JAMA Health Forum*.

e-Table 11: Adjusted Differential Changes in Outcomes, Sensitivity Analysis with Alternative Comparative Interrupted Time Series Specification Allowing for Differential Pre-Trends

e-Table 11 presents the main estimates from a sensitivity analysis using a comparative interrupted time series specification to allow for differential pre-trends between groups.

Variable	Use in First Quarter of Study Period (7/18/17 – 10/15/17)		Group-Specific Pre-Recall Trends (7/18/17 – 7/12/18)[a]		Differential Level Change in First Quarter of Valsartan Recall (7/13/2018 – 10/10/2018)[a]			Differential Trend Change after First Losartan and Irbesartan Recalls (10/11/2018 – 1/3/2019)[a]	
	Users of Recalled ARBs at Baseline	Users of Comparison Drugs at Baseline	Users of Recalled ARBs at Baseline	Users of Comparison Drugs at Baseline	Coef. (95% CI)	p-value	Rel. Change[b]	Coef. (95% CI)	p-value
Mean PDC (p.p.) per patient-quarter	86.0	85.0	0.67	0.54	0.23 (-0.10, 0.57)	0.169	0.27%	-0.36 (-0.5, -0.22)	<0.001
% with Rx. Switch	3.9	1.5	-0.11	0.17	6.72 (6.53, 6.91)	<0.001	172%	-1.2 (-1.2, -1.1)	<0.001
% with Rx. Gap ≥30 days	9.7	10.5	-0.30	-0.25	0.03 (-0.3, 0.4)	0.857	0.31%	0.21 (0.05, 0.38)	0.010
Mean days between fills	10.9	11.9	0.26	0.42	0.27 (-0.12, 0.58)	0.173	2.5%	0.40 (0.23, 0.56)	<0.001
Mean Payer Rx. Spending per patient-quarter	124.1	65.1	-0.04	-1.8	-5.96 (-8.4, -3.5)	<0.001	-4.8%	-0.24 (-1.2, 0.70)	0.618
Mean OOP Rx. Spending per patient-quarter	25.7	191	-0.007	-0.32	-0.50 (-1, -0.003)	0.052	-1.9%	-0.53 (-0.71, -0.35)	<0.001
Generic fills	1.2	1.3	-0.011	-0.009	0.03 (0.02, 0.05)	<0.001	2.5%	0.01 (0.005, 0.01)	<0.001
Rejected fills	0.062	0.060	0.001	0.0007	0.004 (0.0025, 0.004)	<0.001	6.4%	-0.001 (-0.001, -0.0008)	<0.001

Abbreviations: ARB = angiotensin-II-receptor blocker; PDC = proportion of days covered; p.p.=percentage points; Coef. = coefficient; CI = confidence interval; Rel. = relative

a. From comparative interrupted time series model for changes in outcomes in the quarter of valsartan's recall (level change) and linear changes in quarterly trajectory in all subsequent quarters (trend change). Models were adjusted for age group (19-20, 20-29, 30-39, 40-49, 50-64, 65-79, ≥80 years), female gender, pre-recall fill location (retail, long-term-care, mail-order, or unknown/mixed), pre-recall anti-hypertension medication use category (1, 2, 3, or ≥4 drug classes), pre-recall PDC category (<50, 51-79, ≥80 p.p.), primary prescriber specialty, U.S. geographic region, patient metropolitan residential location and insurance type (Medicare, Third party, Medicaid fee-for-service, or Cash/self-pay).

b. Relative changes were calculated as the level change estimate, divided by the use in the first pre-recall quarter (7/18/17 – 10/15/17).

© 2025 Callaway Kim K et al. *JAMA Health Forum*.

e-Table 12: Descriptive Characteristics of Recalled ARB Users, by Post-Recall Switching Status

e-Table 12 presents descriptive statistics on characteristics of recalled ARB users, by switching status and timing of switches post-recall.

Variable	No switch	Switch in First Quarter Post-Recall	Std. Diff.	Switch After First Quarter	Std. Diff.
	10,798,395 (100)	1,704,525 (100)		1,252,331 (100)	
Most-recent Pre-Recall Fill					
Valsartan	1,391,611 (12.9)	1,512,782 (88.8)	2.3296	243,137 (19.4)	0.1781
Irbesartan	501,868 (4.6)	16,972 (1.0)	-0.2219	152,598 (12.2)	0.2740
Losartan	8,904,916 (82.5)	174,771 (10.3)	-2.0994	856,596 (68.4)	-0.3312
Median (IQR) age in 2018	65 (56,74)	67 (58,75)	0.1138	66 (57,74)	0.0130
Patient age in 2018			0.1961		0.1642
<20	5,844 (0.1)	714 (0)		511 (0)	
20-29	64,813 (0.6)	6,824 (0.4)		5,489 (0.5)	
30-39	310,091 (3)	33,859 (2)		29,812 (2.5)	
40-49	1,010,453 (9.6)	127,601 (7.7)		110,293 (9.1)	
50-64	3,732,677 (34.6)	58,538 (32.8)		438,525 (35.0)	
65-79	4,160,380 (38.5)	712,767 (41.8)		511,089 (40.8)	
≥80	1,514,137 (14.0)	264,222 (15.5)		156,612 (12.5)	
Gender					
Female	5,876,256 (54.4)	940,796 (55.2)	0.0156	716,695 (57.2)	0.0566
Male	4,922,139 (45.5)	763,729 (44.8)		535,636 (42.8)	
Region			0.2095		0.1564
Northeast	1,827,955 (17.0)	339,232 (19.9)		222,950 (17.8)	
Midwest	2,246,503 (20.8)	262,750 (15.4)		210,660 (16.8)	
South	4,312,067 (39.9)	785,399 (46.1)		583,281 (46.6)	
West	2,059,951 (19.1)	257,152 (15.1)		203,081 (16.2)	
Missing/unknown	341,919 (3.2)	59,992 (3.5)		32,359 (2.6)	
Patient residential location based on 3-level ZIP code			0.0000		0.0510
<50% metropolitan by population	1,116,110 (10.3)	166,415 (9.8)		107,230 (8.6)	
≥50% metropolitan by population[a]	8629,956 (79.9)	1,361,349 (79.9)		1,026,630 (82.0)	
Missing/unknown	1,052,329 (9.7)	176,761 (10.4)		118,471 (9.5)	
Median (IQR) pre-recall PDC	90.5 (75.9,97.1)	93.1 (81.0,98.0)	0.1873	92 (78.7,97.4)	0.1023
Pre-recall PDC category			0.1661		0.0904
Low (<50 p.p.)	524,031 (4.9)	51,504 (3.0)		45,651 (3.6)	
Moderate (51-79 p.p.)	2,796,874 (25.9)	350,888 (20.6)		288,952 (23.1)	
High (≥80 p.p.)	7,477,490 (69.2)	1,302,133 (76.4)		917,728 (73.3)	
Most common fill location pre-recall			0.0315		0.1588
Retail pharmacy	9,118,706 (84.4)	1,416,544 (83.1)		1,091,983 (87.2)	
Mail-order pharmacy	1,158,839 (10.7)	198,054 (11.6)		109,512 (8.7)	
Long term care facility	90,355 (0.8)	12,693 (0.7)		5,771 (0.5)	
Unknown/mixed/missing	430,495 (4.0)	77,234 (4.5)		45,065 (3.6)	

© 2025 Callaway Kim K et al. *JAMA Health Forum*.

Insurance type on last pre-recall fill			0.1237		0.0882
Medicare[b]	4,720,710 (43.8)	23,596 (48.3)		567,795 (45.3)	
Third-Party[c]	5787,134 (53.6)	856,079 (50.2)		662,926 (52.9)	
Medicaid fee-for-service	117,265 (1.1)	11,884 (0.7)		10,743 (0.9)	
Cash/self-pay	163,286 (1.5)	12,966 (0.8)		10,867 (0.9)	
Primary prescriber specialty pre-recall[d]			0.2228		.NA
Internal medicine or other general practice	7,141,259 (66.1)	1,115,860 (65.5)		848,204 (67.7)	
Physician's assistant or nurse practitioner	1,700,924 (15.8)	242,976 (14.3)		183,842 (14.7)	
Cardiovascular disease	1,172,168 (10.9)	224,760 (13.2)		131,908 (10.5)	
Nephrology	191,885 (1.8)	35,014 (2.1)		22,223 (1.8)	
Diabetes, endocrine, or other metabolic disease	118,048 (1.1)	18,537 (1.1)		15,082 (1.2)	
Other specialty	397,743 (3.7)	57,669 (3.4)		43,928 (3.5)	
Missing/unknown	76,368 (0.7)	9,709 (0.6)		7,144 (0.6)	
Pre-recall anti-hypertension medication use category			0.1045		0.0382
1 drug class	3,856,895 (35.7)	543,618 (31.9)		434,677 (34.7)	
2 drug classes	3,714,786 (34.4)	71,239 (33.5)		427,528 (34.1)	
3 drug classes	2,335,013 (21.6)	402,671 (23.6)		275,596 (22.0)	
≥4 drug classes	891,701 (8.3)	186,997 (11.0)		114,530 (9.1)	
Pre-recall use of other anti-hypertension classes[e]					
Calcium channel blockers	3,495,141 (32.4)	625,711 (36.7)	0.0914	433,435 (34.6)	0.0475
Diuretics	3,163,640 (29.3)	527,585 (31.0)	0.0361	362,043 (28.9)	-0.0085
Adrenergic blockers	4,180,692 (38.7)	738,049 (43.4)	0.0933	497,313 (39.7)	0.0204
Another renin-angiotensin agent	24,414 (0.2)	5,606 (0.3)	0.0195	2,909 (0.2)	0.0013
Other drug class	299,641 (2.8)	65,607 (3.8)	0.0601	40,782 (3.3)	0.0282

Abbreviations: ARB = angiotensin-II-receptor blocker; Std. Diff. = standardized difference; PDC= proportion of days covered; p.p.= percentage points; IQR = interquartile range

a. Areas were defined as predominantly metropolitan if ≥50% of the population resided in ZIP codes classified as 2010 Rural Urban Commuting Areas "1" (Metropolitan area core), "2" (Metropolitan area high commuting) or "3" (Metropolitan area low commuting).
b. Medicare insurance type included both Medicare and Medicare Part D.
c. Third party insurance included coverage from an employer, Medicaid Managed Care, and individual market plans. We did not have access to reliable variables to distinguish between these types.
d. Patients were assigned a single primary prescriber based on the most fills in the pre-recall period (7/18/2017 – 7/12/2018). See e-Table 1 in Supplement for codes used to define each prescriber specialty.
e. Specific drugs included in each anti-hypertension class can be found in e-Table 1 in the Supplement.

© 2025 Callaway Kim K et al. *JAMA Health Forum*.

e-Figure 6: Standardized Trends in Medication Use Outcomes for Recalled ARB vs. Comparison Drug Users, Post-Hoc Sensitivity Analysis by Drug[a]

e-Figure 6 presents unadjusted quarterly trends in medication use outcomes for a sensitivity analysis by drug.

A. Mean Proportion of Days Covered (PDC)

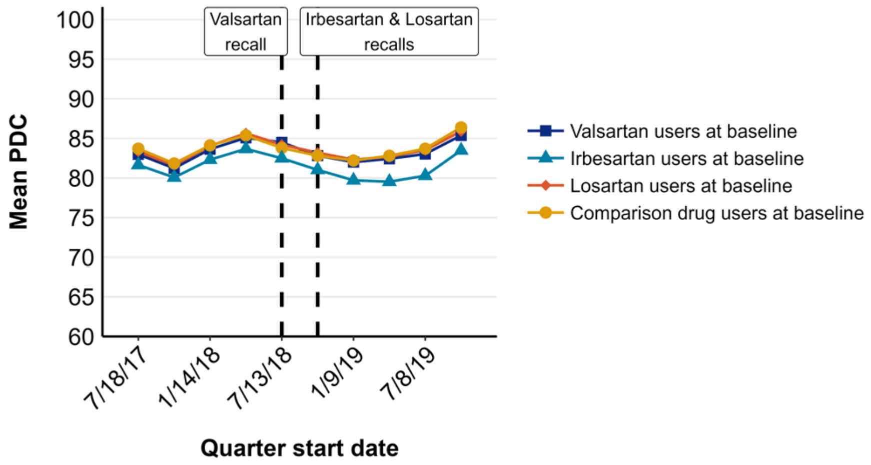

B. Proportion with Rx. Switch

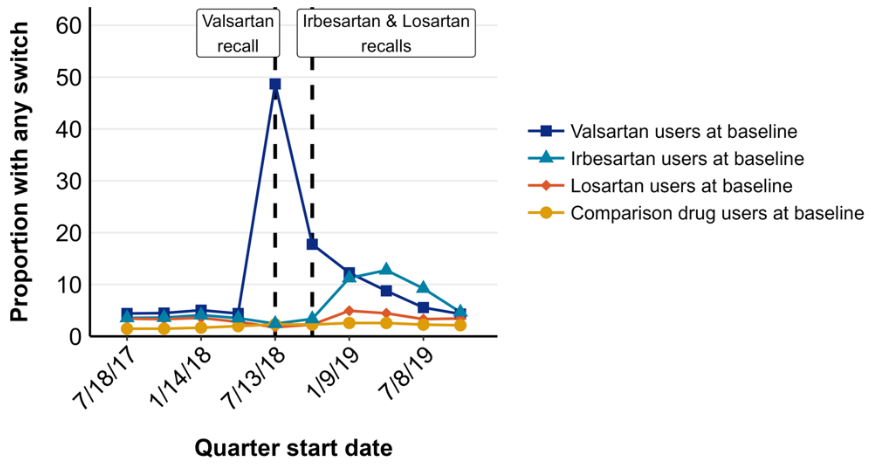

C. Proportion with Rx. Gap ≥30 Days

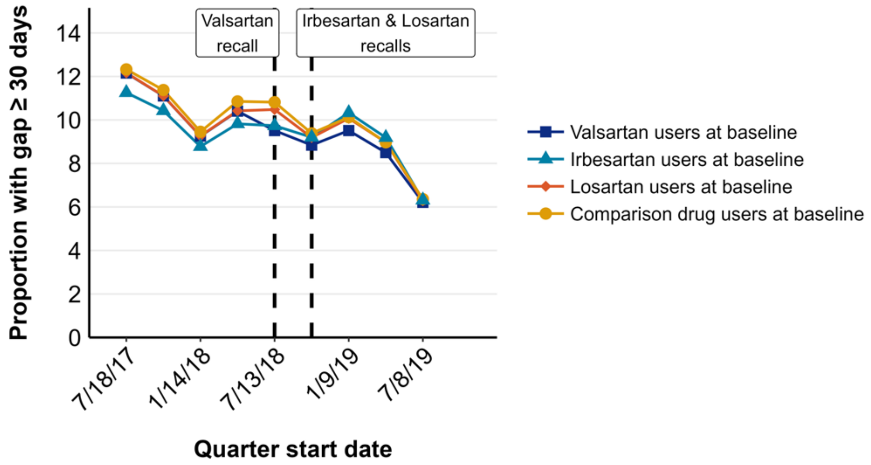

D. Mean Days Between Fills

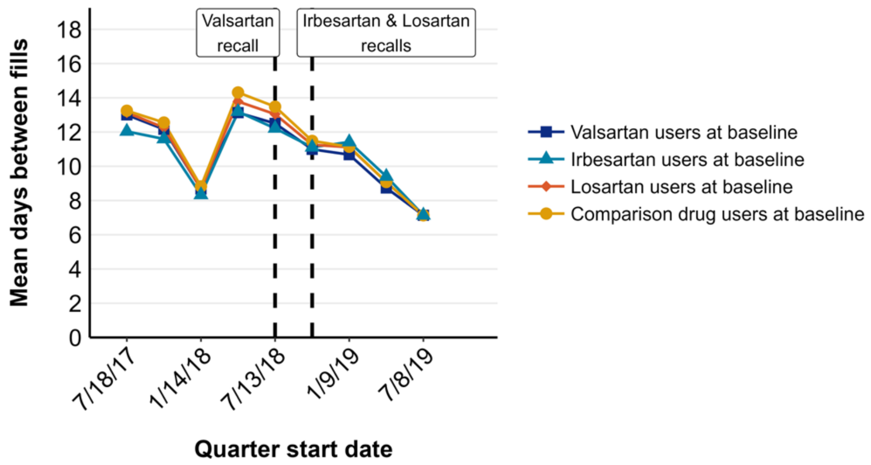

© 2025 Callaway Kim K et al. *JAMA Health Forum*.

a. Quarters were defined as 90-day intervals indexed to the first ARB recall for valsartan on July 13, 2018. Rates were standardized to the population distributions for the following pre-recall characteristics: age group (19-20, 20-29, 30-39, 40-49, 50-64, 65-79, ≥80 years), female gender, pre-recall fill location (retail, long-term-care, mail-order, or unknown/mixed), pre-recall anti-hypertension medication use category (1, 2, 3, or ≥4 drug classes), pre-recall PDC category (<50, 51-79, ≥80 p.p.), primary prescriber specialty, U.S. geographic region, patient metropolitan residential location and insurance type (Medicare, Third party, Medicaid fee-for-service, or Cash/self-pay).

© 2025 Callaway Kim K et al. *JAMA Health Forum*.

e-Figure 7: Standardized Trends in Drug Spending Outcomes for Established ARB vs. Comparison Drug Users, Post-Hoc Sensitivity Analysis by Drug[a]

e-Figure 7 presents unadjusted quarterly trends in drug spending outcomes for a sensitivity analysis by drug.

A. Mean Insurer Spending per Patient

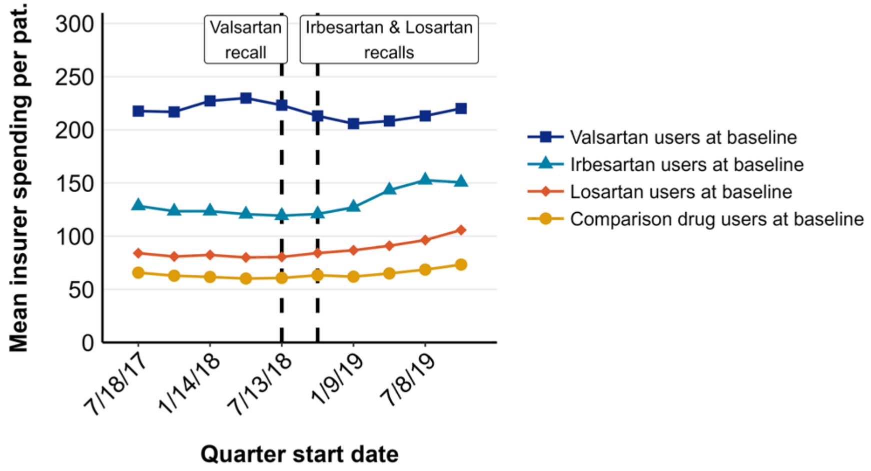

B. Mean Out-of-Pocket Spending per Patient

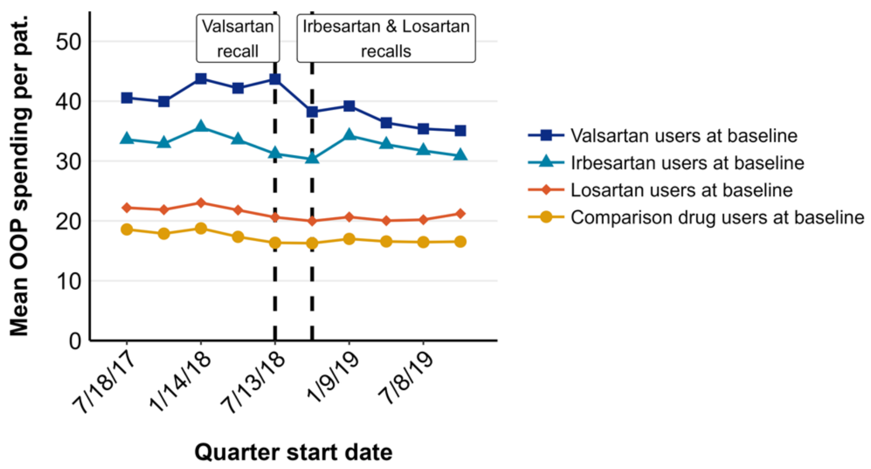

C. Mean Generic Fills per Patient

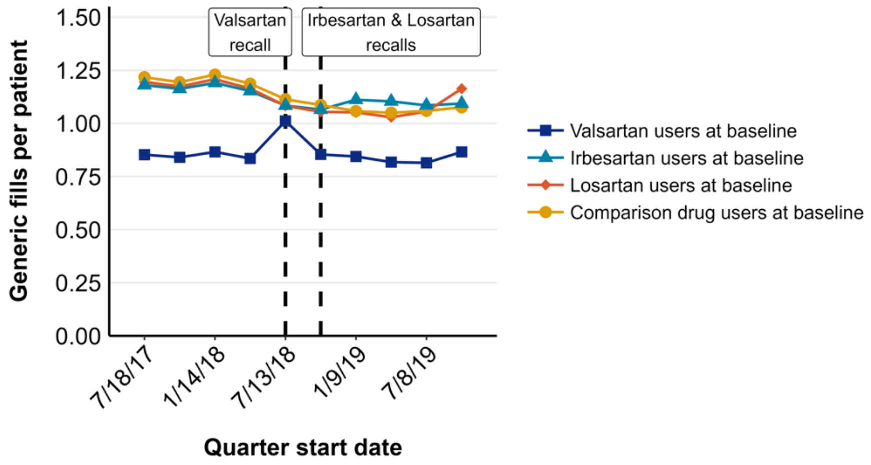

D. Mean Rejected Fills per Patient

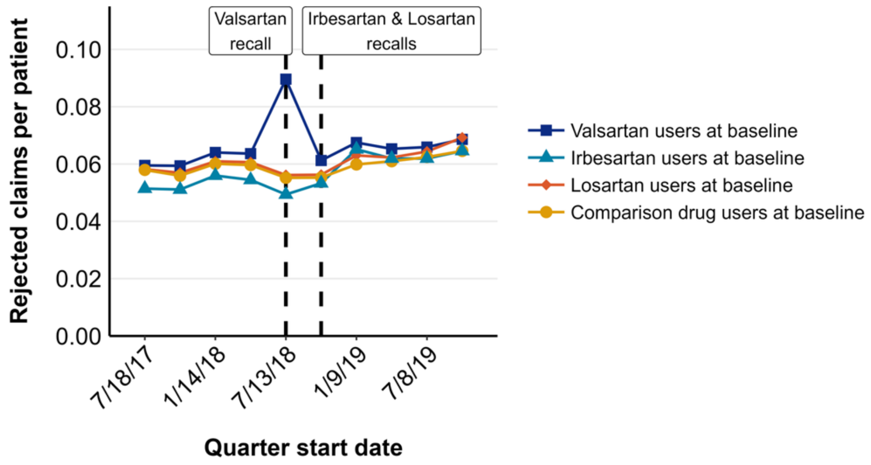

© 2025 Callaway Kim K et al. *JAMA Health Forum*.

a. Quarters were defined as 90-day intervals indexed to the first ARB recall for valsartan on July 13, 2018. Rates were standardized to the population distributions for the following pre-recall characteristics: age group (19-20, 20-29, 30-39, 40-49, 50-64, 65-79, ≥80 years), female gender, pre-recall fill location (retail, long-term-care, mail-order, or unknown/mixed), pre-recall anti-hypertension medication use category (1, 2, 3, or ≥4 drug classes), pre-recall PDC category (<50, 51-79, ≥80 p.p.), primary prescriber specialty, U.S. geographic region, patient metropolitan residential location and insurance type (Medicare, Third party, Medicaid fee-for-service, or Cash/self-pay).

© 2025 Callaway Kim K et al. *JAMA Health Forum*.

e-Table 13: Adjusted Differential Changes in Medication Use Outcomes for Established ARB vs. Comparison Drug Users, Post- vs. Pre-Recall, Post-Hoc Sensitivity Analysis by Drug

e-Table 13 presents results from a difference-in-difference sensitivity analysis for differential changes in medication use outcomes, by drug.

Outcome	Mean Pre-Recall Use per Quarter (7/18/2017 – 7/12/2018)			Difference-in-Difference Estimates[a]								
				First Quarter of Valsartan Recall (7/13/18 – 10/10/18)			First Irbesartan and Losartan Recalls (10/11/18 – 1/8/19)			After First Irbesartan and Losartan Recalls (1/9/19 – 1/3/20)		
	Users of Recalled ARBs at Baseline	Users of Comparison Drugs at Baseline	Diff.	Coef. (95% CI)	p-value	Rel. change [b]	Coef. (95% CI)	p-value	Rel. change[b]	Coef. (95% CI)	p-value	Rel. change[b]
Mean PDC (p.p.) per patient-quarter												
Valsartan users	85.78	84.60	1.19	1.1 (0.9,1.4)	<0.001	1.3%	0.4 (0.2,0.6)	0.001	0.5%	-0.2 (-0.4,0)	0.037	-0.3%
Irbesartan users	87.58	84.60	2.98	-0.1 (-0.3,0.1)	0.479	-0.1%	-0.5 (-0.7, -0.3)	<0.001	-0.6%	-1.8 (-2, -1.6)	<0.001	-2.1%
Losartan users	85.43	84.60	0.83	0.4 (0.2,0.6)	<0.001	0.5%	0.4 (0.2,0.6)	<0.001	0.5%	-0.2 (-0.5,0)	0.027	-0.3%
% With Rx. Switch per patient-quarter												
Valsartan users	4.89	1.59	3.30	44 (43.5,44.5)	<0.001	899.6%	13 (12.8,13.3)	<0.001	266.1%	2.6 (2.5,2.8)	<0.001	53.8%
Irbesartan users	3.88	1.59	2.28	-1.9 (-2, -1.9)	<0.001	-49.5%	-0.8 (-0.9, -0.8)	<0.001	-21.7%	5.8 (5.6,6)	<0.001	149.9%
Losartan users	3.30	1.59	1.71	-2.1 (-2.2, -2.1)	<0.001	-64.3%	-1.6 (-1.6, -1.5)	<0.001	-47.1%	0.2 (0.1,0.2)	<0.001	5.7%
% With Rx. Gap ≥30 days per patient-quarter												
Valsartan users	9.47	10.24	-0.78	-1 (-1.3, -0.8)	<0.001	-10.4%	-0.1 (-0.4,0.2)	0.46	-1.0%	0.1 (-0.2,0.3)	0.497	0.9%
Irbesartan users	8.19	10.24	-2.06	0.2 (0,0.4)	0.059	2.5%	1.4 (1.1,1.6)	<0.001	16.6%	1.9 (1.6,2.1)	<0.001	22.8%
Losartan users	9.53	10.24	-0.71	-0.03 (-0.24,0.18)	0.777	-0.3%	0.2 (0,0.4)	0.116	2.1%	0.4 (0.2,0.6)	0.001	4.2%
Mean days between fills per patient-quarter												

© 2025 Callaway Kim K et al. *JAMA Health Forum*.

Valsartan users	10.46	11.56	-1.09	-0.6 (-0.8, -0.4)	<0.001	-5.6%	0.2 (-0.1,0.4)	0.155	1.5%	0.5 (0.3,0.8)	<0.001	5.0%
Irbesartan users	9.45	11.56	-2.11	-0.18 (-0.38,0.03)	0.079	-1.9%	1.2 (0.9,1.4)	<0.001	11.8%	2.0 (1.8,2.3)	<0.001	21.3%
Losartan users	10.74	11.56	-0.81	-0.2 (-0.39,0)	0.043	-1.8%	0.2 (0,0.4)	0.076	1.9%	0.5 (0.3,0.8)	<0.001	4.9%

Abbreviations: ARB = angiotensin-receptor-II blocker; Rx. = prescription; Diff. = difference; Coef. = coefficient; CI = confidence interval; Rel. = relative

a. Difference-in-difference estimates for the intention-to-treat differential change in outcomes for ARB versus comparison drug users. Models were adjusted for age group (19-20, 20-29, 30-39, 40-49, 50-64, 65-79, ≥80 years), female gender, pre-recall fill location (retail, long-term-care, mail-order, or unknown/mixed), pre-recall anti-hypertension medication use category (1, 2, 3, or ≥4 drug classes), pre-recall PDC category (<50, 51-79, ≥80 p.p.), primary prescriber specialty, U.S. geographic region, patient metropolitan residential location and insurance type (Medicare, Third party, Medicaid fee-for-service, or Cash/self-pay).
b. Relative changes were calculated as the difference-in-difference estimate, divided by the mean quarterly use among baseline ARB users in the pre-recall period (7/18/2017 – 7/12/2018).

© 2025 Callaway Kim K et al. *JAMA Health Forum*.

e-Table 14: Adjusted Differential Changes in Medication Use Outcomes for Established ARB vs. Comparison Drug Users, Post- vs. Pre-Recall, Post-Hoc Sensitivity Analysis by Drug

e-Table 14 presents results from a difference-in-difference sensitivity analysis for differential changes in drug spending outcomes, by drug.

Outcome	Mean Pre-Recall Use per Quarter (7/18/2017 – 7/12/2018)			Difference-in-Difference Estimates[a] First Quarter of Valsartan Recall (7/13/18 – 10/10/18)			First Irbesartan and Losartan Recalls (10/11/18 – 1/8/19)			After First Irbesartan and Losartan Recalls (1/9/19 – 1/3/20)		
	Users of Recalled ARBs at Baseline	Users of Comparison Drugs at Baseline	Diff.	Coef. (95% CI)	p-value	Rel. change[b]	Coef. (95% CI)	p-value	Rel. change[b]	Coef. (95% CI)	p-value	Rel. change[b]
Mean Payer Rx. Spending per patient-quarter												
Val. users	238.21	61.55	176.65	4.6 (-1.1,10.2)	0.111	1.9%	-9.3 (-14.7, -4)	<0.001	-3.9%	-14.9 (-18.4, -11.5)	<0.001	-6.3%
Irb. users	127.07	61.55	65.51	-3.7 (-6.7, -0.7)	0.017	-2.9%	-4.5 (-7.5, -1.6)	0.002	-3.6%	17.6 (15.5,19.7)	<0.001	13.8%
Los. users	86.36	61.55	23.81	0.1 (-1.4,1.6)	0.88	0.1%	1.2 (-0.2,2.7)	0.096	1.5%	8.5 (7.4,9.6)	<0.001	9.9%
Mean OOP Rx. Spending per patient-quarter												
Val. users	39.15	18.41	20.74	3.9 (3.2,4.6)	<0.001	9.9%	-1.4 (-2, -0.7)	<0.001	-3.5%	-2.7 (-3.2, -2.3)	<0.001	-7.0%
Irb. users	32.06	18.41	13.65	-1.1 (-1.6, -0.5)	<0.001	-3.3%	-1.8 (-2.3, -1.3)	<0.001	-5.5%	0.6 (0.2,0.9)	0.002	1.7%
Los. users	20.44	18.41	2.03	0.2 (-0.2,0.6)	0.338	0.9%	-0.3 (-0.6,0.1)	0.131	-1.3%	0.2 (0,0.4)	0.11	0.9%
Generic fills per patient-quarter												
Val. users	0.86	1.21	-0.35	0.3 (0.2,0.3)	<0.001	30.4%	0.1 (0.1,0.1)	<0.001	14.4%	0.1 (0.1,0.1)	<0.001	14.7%
Irb. users	1.20	1.21	0	0.001 (-0.01,0.012)	0.776	0.1%	0.01 (0,0.02)	0.056	0.8%	0.08 (0.07,0.08)	<0.001	6.3%

© 2025 Callaway Kim K et al. *JAMA Health Forum*.

Los. users	1.20	1.21	-0.01	-0.01 (-0.02,0)	0.039	-0.9%	-0.01 (-0.02,0)	0.013	-1.1%	0.03 (0.02,0.04)	<0.001	2.6%
Rejected fills per patient-quarter												
Val. users	0.06	0.06	0	0.03 (0.03,0.03)	<0.001	49.9%	0.003 (0.002,0.004)	<0.001	4.9%	0.002 (0.001,0.002)	<0.001	3.1%
Irb. users	0.05	0.06	-0.01	-0.001 (-0.002,0)	0.21	-1.1%	0.003 (0.002,0.004)	<0.001	6.3%	0.007 (0.007,0.008)	<0.001	13.5%
Los. users	0.06	0.06	0	0 (0,0.001)	0.584	0.3%	0.0001 (-0.001,0.001)	0.789	0.1%	0.002 (0.002,0.002)	<0.001	3.3%

Abbreviations: ARB = angiotensin-receptor-II blocker; Rx. = prescription; OOP = out-of-pocket; Diff. = difference; Coef. = coefficient; CI = confidence interval; Rel. = relative; Val. = valsartan; Irb. = irbesartan; Los. = losartan

a. Difference-in-difference estimates for the intention-to-treat differential change in outcomes for ARB versus comparison drug users. Models were adjusted for age group (19-20, 20-29, 30-39, 40-49, 50-64, 65-79, ≥80 years), female gender, pre-recall fill location (retail, long-term-care, mail-order, or unknown/mixed), pre-recall anti-hypertension medication use category (1, 2, 3, or ≥4 drug classes), pre-recall PDC category (<50, 51-79, ≥80 p.p.), primary prescriber specialty, U.S. geographic region, patient metropolitan residential location and insurance type (Medicare, Third party, Medicaid fee-for-service, or Cash/self-pay).

b. Relative changes were calculated as the difference-in-difference estimate, divided by the mean quarterly use among baseline ARB users in the pre-recall period (7/18/2017 – 7/12/2018).

© 2025 Callaway Kim K et al. *JAMA Health Forum*.

e-Table 15: Reasons for Rejected Fills, Post-Hoc Sensitivity Analysis by Drug

e-Table 15 describes listed reasons for rejected fills pre-recall, during the first 90 days after valsartan's first recall, and in the 5 quarters after irbesartan and losartan's first recalls for a post-hoc sensitivity analysis by study medication (valsartan, irbesartan, losartan).

	No. Rejected Fills (% of Rejected)		
Reason for Rejected Fill	Pre-Recall (7/18/17 – 7/12/18)	First Quarter of Valsartan Recall (7/13/18 – 10/10/18)	After First Irbesartan and Losartan Recalls (10/11/18 – 1/3/20)
Valsartan users at baseline			
Refill restriction	363,549 (48.7%)	105,975 (38.4%)	572,153 (64.0%)
Step edit	222,708 (29.8%)	51,937 (18.8%)	125,254 (14.0%)
Missing or invalid information on claim	64,772 (8.7%)	3,545 (19.4%)	97,509 (10.9%)
Product not covered	41,291 (5.5%)	41,687 (15.1%)	9,137 (5.5%)
Plan limitations exceeded	24,498 (3.3%)	14,973 (5.4%)	30,427 (3.4%)
Prior authorization required	25,775 (3.5%)	7,091 (2.6%)	16,440 (1.8%)
Distribution limitation	3,105 (0.4%)	871 (0.3%)	3,249 (0.4%)
Missing reject code	550 (0.1%)	4 (<0.01%)	1 (<0.01%)
Irbesartan users at baseline			
Refill restriction	75,546 (54.1%)	20,401 (62.4%)	118,320 (64.4%)
Step edit	41,277 (29.6%)	7,918 (24.2%)	25,587 (13.9%)
Missing or invalid information on claim	9,422 (6.7%)	2,189 (6.7%)	18,777 (10.2%)
Product not covered	6,144 (4.4%)	795 (2.4%)	8,091 (4.4%)
Plan limitations exceeded	5,436 (3.9%)	1,138 (3.5%)	11,068 (6.0%)
Prior authorization required	1,132 (0.8%)	155 (0.5%)	1,148 (0.6%)
Distribution limitation	490 (0.4%)	106 (0.3%)	749 (0.4%)
Missing reject code	153 (0.1%)	NA	NA
Losartan users at baseline			
Refill restriction	1,234,436 (54.0%)	347,138 (62.5%)	1,879,204 (68.5%)
Step edit	725,684 (31.8%)	1359,260 (25.1%)	409.641 (14.9%)
Missing or invalid information on claim	175,133 (7.7%)	41,270 (7.4%)	69,382 (9.8%)
Product not covered	58,796 (2.6%)	11,102 (2.0%)	86,424 (3.1%)
Plan limitations exceeded	62,999 (2.8%)	13,018 (2.3%)	74,303 (2.7%)
Prior authorization required	13,267 (0.6%)	1,720 (0.3%)	15,267 (0.6%)
Distribution limitation	11,587 (0.5%)	2,268 (0.4%)	10,880 (0.4%)
Missing reject code	2,128 (0.1%)	11 (<0.01%)	NA

Abbreviations: No. = number

© 2025 Callaway Kim K et al. *JAMA Health Forum*.

e-Table 16: Adjusted Differential Changes in Proportion with Rx. Switch for Established ARB vs. Comparison Drug Users, Post- vs. Pre-Recall, Post-Hoc Sensitivity Analysis by Baseline MPR, Region, Prescriber Specialty, and Insurance Type

e-Table 16 presents results from a difference-in-difference sensitivity analysis for differential changes in medication switches, by pre-recall medication possession ratio, region, prescriber specialty, and insurance type.

Outcome	Mean Pre-Recall Use per Quarter, (7/18/2017 – 7/12/2018)			Difference-in-Difference Estimates[a]					
				First Quarter of Valsartan Recall (7/13/18 – 10/10/18)			After Irbesartan and Losartan Recalls (10/11/18 – 1/8/19)		
	Users of Recalled ARBs at Baseline	Users of Comparison Drugs at Baseline	Diff.	Coef. (95% CI)	p-value	Rel. change[b]	Coef. (95% CI)	p-value	Rel. change[b]
Pre-recall PDC category									
Low (<50 p.p.)	2.2	0.9	1.3	5.72 (5.53, 5.91)	<0.001	259.1%	1.08 (1.0, 1.17)	<0.001	50.0%
Moderate (51-79 p.p.)	3.5	1.3	2.2	6.48 (6.32, 6.65)	<0.001	185.7%	0.57 (0.48, 0.66)	<0.001	17.1%
High (≥80 p.p.)	3.8	1.7	2.1	9.31 (9.10, 9.51)	<0.001	244.7%	1.41 (1.31, 1.50)	<0.001	36.8%
Region									
Northeast	3.3	1.6	1.7	10.67 (10.45, 10.88)	<0.001	324.2%	1.67 (1.58, 1.77)	<0.001	51.5%
Midwest	3.5	1.2	2.3	5.86 (5.71, 6.07)	<0.001	165.7%	0.31 (0.19, 0.42)	<0.001	8.6%
South	4.1	1.9	2.2	9.51 (9.32, 9.69)	<0.001	231.7%	1.63 (1.49, 1.76)	<0.001	39.0%
West	3.7	1.5	2.2	6.24 (5.95, 6.51)	<0.001	167.6%	0.53 (0.41, 0.66)	<0.001	13.5%
Primary prescriber specialty pre-recall									
Internal medicine	3.3	1.5	1.8	8.23 (8.07, 8.39)	<0.001	248.4%	1.28 (1.21, 1.34)	<0.001	39.3%
NP/PA	4.3	1.4	2.9	8.70 (8.47, 8.94)	<0.001	202.3%	1.56 (1.46,1.65)	<0.001	37.2%
Cardiovascular disease	5.2	2.4	2.8	6.86 (6.60, 7.12)	<0.001	132.7%	0.33 (0.23, 0.43)	0.011	5.8%
All other specialties	3.9	2.0	1.9	9.41 (9.15, 9.67)	<0.001	241.0%	0.16 (0.03, 0.28)	<0.001	5.2%
Insurance type on last pre-recall fill									
Medicare	3.7	1.9	1.8	9.49 (9.27, 9.72)	<0.001	256.8%	1.63 (1.51, 1.74)	<0.001	43.2%
Third-Party	3.7	1.4	2.3	7.81 (7.57, 8.03)	<0.001	210.8%	0.89 (0.80, 0.98)	<0.001	24.3%

© 2025 Callaway Kim K et al. *JAMA Health Forum*.

Medicaid fee-for-service	5.8	1.5	4.3	2.54 (2.31, 2.77)	<0.001	43.1%	-1.52 (-1.64, -1.41)	<0.001	-25.8%
Cash/self-pay	3.9	0.9	3.0	3.42 (3.22, 3.61)	<0.001	87.1%	-0.68 (-0.76, -0.59)	<0.001	-17.9%

Abbreviations: ARB = angiotensin-receptor-II blocker; Diff. = difference; Coef. = coefficient; CI = confidence interval; Rel. = relative

a. Difference-in-difference estimates for the intention-to-treat differential change in outcomes for ARB versus comparison drug users. Estimates within each defined group were calculated from a triple-difference model which allowed for different effects within each level of the given category. Models were adjusted for age group (19-20, 20-29, 30-39, 40-49, 50-64, 65-79, ≥80 years), female gender, pre-recall fill location (retail, long-term-care, mail-order, or unknown/mixed), pre-recall anti-hypertension medication use category (1, 2, 3, or ≥4 drug classes), and patient metropolitan residential location, plus the grouping variables above (pre-recall PDC category, region, prescriber specialty, and insurance type).

b. Relative changes were calculated as the difference-in-difference estimate, divided by the mean quarterly use among baseline ARB users in the pre-recall period (7/18/2017 – 7/12/2018).

© 2025 Callaway Kim K et al. *JAMA Health Forum*.
