# Supplementary material for: Adherence, Switches, and Drug Spending After Angiotensin Receptor Blocker Recalls and Shortages
Source: JAMA Health Forum. 2025 Nov 26;6(11):e254078. doi: 10.1001/jamahealthforum.2025.4078 (PMC12658656; doi:10.1001/jamahealthforum.2025.4078)
Supplement: Supplement 2. — Data Sharing Statement [file jamahealthforum-e254078-s002.pdf]

Data Sharing Statement

Callaway Kim. Adherence, Switches, and Drug Spending After Angiotensin Receptor Blocker Recalls and Shortages. *JAMA Health Forum*. Published November 26, 2025. doi:10.1001/jamahealthforum.2025.4078

Data
Data available: No

Additional Information
Explanation for why data not available: Patient-level IQVIA data cannot be shared per our DUA. Aggregated trends may be made available upon reasonable request.
